# Guidance framework to apply best practices in ecological data analysis: lessons learned from building Galaxy-Ecology

**DOI:** 10.1093/gigascience/giae122

**Published:** 2025-02-12

**Authors:** Coline Royaux, Jean-Baptiste Mihoub, Marie Jossé, Dominique Pelletier, Olivier Norvez, Yves Reecht, Anne Fouilloux, Helena Rasche, Saskia Hiltemann, Bérénice Batut, Eléaume Marc, Pauline Seguineau, Guillaume Massé, Alan Amossé, Claire Bissery, Romain Lorrilliere, Alexis Martin, Yves Bas, Thimothée Virgoulay, Valentin Chambon, Elie Arnaud, Elisa Michon, Clara Urfer, Eloïse Trigodet, Marie Delannoy, Gregoire Loïs, Romain Julliard, Björn Grüning, Yvan Le Bras, The Galaxy-E community

**Affiliations:** UMR8067 Biologie des Organismes et Ecosystèmes Aquatiques (BOREA, MNHN-CNRS-SU-IRD-UCN-UA), Sorbonne Université, Station Marine de Concarneau, 29900 Concarneau, France; Pôle national de données de biodiversité, UAR2006 PatriNat (OFB-MNHN-CNRS-IRD), Muséum National d'Histoire Naturelle, Station Marine de Concarneau, 29900 Concarneau, France; Centre d’Écologie et des Sciences de la Conservation (UMR7204 CESCO, MNHN-CNRS-SU), Muséum National d'Histoire Naturelle, Sorbonne Université, Centre National de la Recherche Scientifique, 75005 Paris, France; Data Terra, Centre National de la Recherche Scientifique, 29200 Brest, France; UMR DECOD (Ifremer-Agrocampus Ouest-INRAE), 56100 Lorient, France; Pôle National de Données de Biodiversité, UAR2006 PatriNat (OFB-MNHN-CNRS-IRD), Fondation pour la Recherche sur la Biodiversité, Muséum national d'Histoire naturelle, 75005 Paris, France; Institute of Marine Research, 5817 Bergen, Norway; Institut français de recherche pour l'exploitation de la mer (Ifremer), 29200 Brest, France; Simula Research Laboratory, 0164 Oslo, Norway; Department of Pathology and Clinical Bioinformatics, Erasmus Medical Center, 3000 CA Rotterdam, The Netherlands; Institute of Pharmaceutical Sciences, Faculty of Chemistry and Pharmacy, University of Freiburg, 79104 Freiburg, Germany; Institut Français de Bioinformatique, CNRS UAR3601, 91042 Évry, France; Mésocentre, Clermont-Auvergne, Université Clermont Auvergne, 63000 Clermont-Ferrand, France; Institut de Systématique Evolution, Biodiversité (UMR7205 ISYEB, MNHN-CNRS-SU-EPHE), Département Origines et Évolution, Muséum national d'Histoire naturelle, 75005 Paris, France; Institut de Systématique Evolution, Biodiversité (UMR7205 ISYEB, MNHN-CNRS-SU-EPHE), Département Origines et Évolution, Station Marine de Concarneau, 29900 Concarneau, France; Institut de Systématique Evolution, Biodiversité (UMR7205 ISYEB, MNHN-CNRS-SU-EPHE), Département Origines et Évolution, Muséum national d'Histoire naturelle, 75005 Paris, France; Institut de Systématique Evolution, Biodiversité (UMR7205 ISYEB, MNHN-CNRS-SU-EPHE), Département Origines et Évolution, Station Marine de Concarneau, 29900 Concarneau, France; UMR LOCEAN (CNRS-SU-IRD-MNHN), Centre National de la Recherche Scientifique, Station Marine de Concarneau, 29900 Concarneau, France; Muséum National d'Histoire Naturelle, Station Marine de Concarneau, 29900 Concarneau, France; Institut français de recherche pour l'exploitation de la mer (Ifremer), 29200 Brest, France; Université Claude Bernard Lyon 1, 69000 Lyon, France; Centre d’Écologie et des Sciences de la Conservation (UMR7204 CESCO, MNHN-CNRS-SU), Muséum National d'Histoire Naturelle, Sorbonne Université, Centre National de la Recherche Scientifique, 75005 Paris, France; UMR8067 Biologie des Organismes et Ecosystèmes Aquatiques (BOREA, MNHN-CNRS-SU-IRD-UCN-UA), Muséum national d'Histoire naturelle, 75005 Paris, France; Centre d’Écologie et des Sciences de la Conservation (UMR7204 CESCO, MNHN-CNRS-SU), Muséum National d'Histoire Naturelle, Sorbonne Université, Centre National de la Recherche Scientifique, 75005 Paris, France; UAR2006 PatriNat (OFB-MNHN-CNRS-IRD), Muséum national d'Histoire naturelle, 75005 Paris, France; Centre d’Écologie et des Sciences de la Conservation (UMR7204 CESCO, MNHN-CNRS-SU), Muséum National d'Histoire Naturelle, 29900 Concarneau, France; Université de Montpellier, 34000 Montpellier, France; Muséum National d'Histoire Naturelle, Station Marine de Concarneau, 29900 Concarneau, France; Pôle national de données de biodiversité, UAR2006 PatriNat (OFB-MNHN-CNRS-IRD), Muséum National d'Histoire Naturelle, Station Marine de Concarneau, 29900 Concarneau, France; Institut des Sciences de la Mer de Rimouski, Université du Québec à Rimouski, Rimouski G5L 2Z9, Québec, Canada; Pôle national de données de biodiversité, UAR2006 PatriNat (OFB-MNHN-CNRS-IRD), Muséum National d'Histoire Naturelle, Station Marine de Concarneau, 29900 Concarneau, France; Université de Bretagne Occidentale, 29200 Brest, France; Centre d’Écologie et des Sciences de la Conservation (UMR7204 CESCO, MNHN-CNRS-SU), Muséum National d'Histoire Naturelle, 29900 Concarneau, France; Université de Bretagne Occidentale, 29200 Brest, France; Centre d’Écologie et des Sciences de la Conservation (UMR7204 CESCO, MNHN-CNRS-SU), Muséum National d'Histoire Naturelle, Sorbonne Université, Centre National de la Recherche Scientifique, 75005 Paris, France; Centre d’Écologie et des Sciences de la Conservation (UMR7204 CESCO, MNHN-CNRS-SU), Muséum National d'Histoire Naturelle, Sorbonne Université, Centre National de la Recherche Scientifique, 75005 Paris, France; Centre d’Écologie et des Sciences de la Conservation (UMR7204 CESCO, MNHN-CNRS-SU), Muséum National d'Histoire Naturelle, Sorbonne Université, Centre National de la Recherche Scientifique, 75005 Paris, France; Bioinformatics Group, Department of Computer Science, Albert-Ludwigs-University Freiburg, 79110 Freiburg, Germany; Pôle national de données de biodiversité, UAR2006 PatriNat (OFB-MNHN-CNRS-IRD), Muséum National d'Histoire Naturelle, Station Marine de Concarneau, 29900 Concarneau, France

**Keywords:** biodiversity, reproducible analyses, Galaxy, best practices, atomization, generalization, workflows, ecoinformatics

## Abstract

Numerous conceptual frameworks exist for best practices in research data and analysis (e.g., Open Science and FAIR principles). In practice, there is a need for further progress to improve transparency, reproducibility, and confidence in ecology. Here, we propose a practical and operational framework for researchers and experts in ecology to achieve best practices for building analytical procedures from individual research projects to production-level analytical pipelines. We introduce the concept of atomization to identify analytical steps that support generalization by allowing us to go beyond single analyses. The term atomization is employed to convey the idea of single analytical steps as “atoms” composing an analytical procedure. When generalized, “atoms” can be used in more than a single case analysis. These guidelines were established during the development of the Galaxy-Ecology initiative, a web platform dedicated to data analysis in ecology. Galaxy-Ecology allows us to demonstrate a way to reach higher levels of reproducibility in ecological sciences by increasing the accessibility and reusability of analytical workflows once atomized and generalized.

## Graphical Abstract

**Graphical Abstract:**
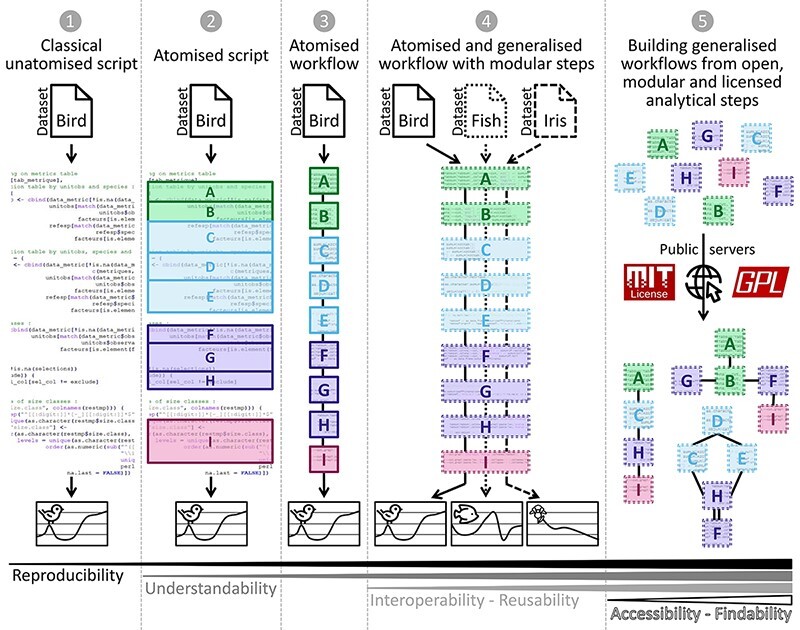
Levels of attainable best practices through the atomization–generalization framework.

## Background

### Ecology’s reproducibility crisis

Research in ecology is increasingly shaped by the availability of novel analytical solutions and statistical tools. Given the ever-growing amount of data available, much attention is often given to the thought process behind statistical analyses to handle different data distributions, pseudo-replication, and sampling biases for instance [1–3]. Despite the high-quality standards required by the scientific community from data access to analysis, the level of complexity of ecological systems makes results difficult to reproduce. The ongoing “reproducibility crisis” has also led researchers to pay closer attention to the quality of analyses to increase confidence in their studies and conclusions [4, 5]. Reproducibility (i.e., different teams and experimental setups obtaining similar results) [6] is one of the main criteria for evaluating robust science and reliable conclusions. The term “reproducibility” is a relative concept and has known various definitions depending on field and context. Reproducibility of analyses (“computational reproducibility”) is defined by Cohen-Boulakia et al. [7] as the ability of distinct analyses to reach to the same conclusion.

In the current context of the global biodiversity crisis, the scientific community needs to use all available data and provide as robust as possible evidence regarding the state and dynamic of ecological systems, from genetic to ecosystem. At the same time, using analytical tools to provide robust evidence can be complex and may require advanced skills that are not widely available across the scientific community [2]. Therefore, operational solutions and methodological guidelines can allow analytical workflows to be more accessible without degrading the scientific quality of analyses and thus promote efficient and broad deployment of best practices.

### Is the ecology community failing to meet best practices?

The first step toward reproducibility is knowing current best practices and recommendations. Among them, the FAIR principles [8], for which the availability of the data and the code used for each published result is an essential criterion, may be key for appropriate management through the data life cycle [9]. The FAIR principles (see also CARE principles [10]) are considered a founding framework to share data along 4 important elements: “Findable” for humans and machines, “Accessible” with a detailed access procedure, “Interoperable” for interaction with other data or applications, and “Reusable” in an identical or different context. In addition to these principles, propositions have been delimited within several thematic communities in ecology to evaluate and enhance best practices application, notably the species distribution modeling communities [11, 12].

Although data accessibility has been substantially improved in ecology during the past decade, sharing analytical scripts and codes remains largely marginal [13–16]. However, even if sharing code is necessary to achieve good computational reproducibility, it is insufficient. Therefore, the utilization of computational workflows has been suggested as a solution for improving computational reproducibility [7, 17] through software such as Snakemake [18–20], Nextflow [21, 22], or Galaxy [23, 24]. A workflow is generally defined as a sequence of distinct computational tasks for a particular objective [25]. As such, a workflow represents the backbone of a single specific analysis. Throughout the analytical procedure, a typical workflow starts with raw data, which can be extracted from several databases or data files and processed through a series of analytical steps. The products resulting from these analytical steps (i.e., the outputs of the computational workflow) can be data files, graphic representations, and any associated metrics.

When properly designed, a certain level of reproducibility can be easily achieved since workflow languages naturally capture the following 4 key elements [7]:

– the specificities of the workflow, the analysis steps, and associated tools;
– the workflow entries, datasets, and parameters;
– the environment and context of the use of the workflow; and
– the results obtained and the outputs of the workflow.

In the original publication of Wilkinson et al. [8], the focus of FAIR principles was mainly on observational data. However, the principles can be applied to software and computational workflows [25, 26]. For instance, a code shared as supplementary material of a non–open access publication could be considered “Interoperable” but is not easily “Findable,” “Accessible,” or “Reusable.” In contrast, a large block of code consisting of several hundred lines, from data preprocessing to final results and graphics, as pictured in the Graphical abstract 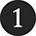, may require efforts to understand and adapt to other kinds of data (“nonreusable”), mainly if annotations or comments are limited. Similarly, an analytical procedure shared without indicating the versions of hardware, software, and packages has a low chance of producing identical outputs, making it less reproducible. These issues may harm the scientific community by preventing fully transparent communication among users about knowledge production and practice comparison. They can also be detrimental to individual authors, when they need to update or run new analyses.

### Impact on ecology research

The efficiency of the scientific process is greatly affected by the lack of computational reproducibility and FAIRness of analytical procedures. The adoption of FAIR practices was estimated to save 10.2 billion euros per year in Europe [27–29]. Moreover, consistent application of reproducibility and FAIR principles will improve trust in research studies and scientific reports [30–32].

The widespread use of computational languages to process large-scale data and analyze complex systems has been a major advance in studying the ecosphere at any spatiotemporal scale [33, 34]. However, the ever-growing technical and programming skills required to take advantage of such computational solutions by the scientific community raise new challenges [35–37]. The use of increasingly complex analytical solutions, paired with different approaches or programming languages, creates barriers to uptake and challenges for peer review. Indeed, many ecologists have acquired their programming skills through self-study or through courses that combine instruction in statistics and ecological principles with an introduction to programming. This learning process does not inherently compromise the quality of the analyses and results; however, it may lead to inappropriate coding habits. As a response to this situation, adequate training was identified by life science researchers [38–40], as it would help involve more people in the understanding of current analytical solutions and benefit to scientific cooperation [41, 42]. Research is typically structured through a highly competitive organization, with a potentially detrimental effect on scientific knowledge [43]. Instead, fostering collaboration and collective intelligence by promoting transparent sharing of analytical procedures would offer more persistent and robust ways to achieve actionable science [44]. Such efforts would be of paramount importance in environmental sciences and the conservation of biodiversity by providing governance and guiding actions with increasingly robust evidence [45].

### Are there simple and ready-to-use solutions?

In this article, we aim to promote the reuse of existing concepts and solutions as pillars toward better practices for ecological analyses by providing a streamlined framework. We believe the atomization–generalization framework presented in the second part of this article represents an operational and actionable path for researchers and experts to attain levels of best practices (e.g., reproducibility, FAIR, open science, R compendium) [46] with no more investment than they are able or willing to provide [47]. Atomization is used to refer to the identification of distinct analytical steps, each constituting an analytical procedure. It is a nonstandard term introduced in this article to convey the idea of analytical “atoms.” As for atom particles that etymologically correspond to “indivisible” but are composed of subatomic particles, an analytical atom represents a single analytical step composed of several functions. Generalization involves the alteration of an analytical step to enlarge its applicability in diverse contexts and for diverse purposes. Therefore, generalization cannot be efficiently achieved without prior atomization.

Atomization and generalization are central organizing principles in the design of the Galaxy-Ecology (Galaxy-E) initiative (see section "Entering a new dimension: the Galaxy-E initiative example"). Galaxy-E is a demonstration platform for applying best practices such as the FAIR principles and computational reproducibility for analytical procedures in ecology. Hence, this review article is partly Galaxy-oriented, not to present the platform as a prescriptive solution but to give an operational example of the best practices it helps to achieve.

## Main Text

### Guidelines for best practices

#### Atomization: what is it and why?

Atomization refers to dividing an analytical procedure into several specific steps (“atoms”; Graphical abstract 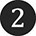), generating a suite of elementary analytical steps as pictured in the Graphical abstract 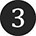. For instance, in a maximally atomized workflow, each small step would be conducted by its own bespoke function. Breaking down the analytical process into atoms functioning as building blocks allows for better understanding, modularity, and visibility of the analytical flow. It permits making it more accessible to a broader audience or facilitating the peer-review process. Indeed, an extended 1-block code that imports raw data, makes preprocessing steps (e.g., filter, formatting), conducts analyses (e.g., distribution study, modeling), and performs final representations of results (e.g., maps, plots) can be challenging to understand and reuse by others or even the same person after some time.

McIntire et al. [48] described the PERFICT approach (Prediction, Evaluation, Reusability, Free access, Interoperability, Continuous workflows, and routine Tests) to set a new foundation for models in predictive ecology. This can be applied more generally to the analytical procedure in ecology and biodiversity. In their article, McIntire and collaborators make an analogy between code development and Lego construction, similar to our definition of atomization. Functions are a workflow’s most fundamental analytical steps and can be seen as modular pieces, like single pieces of Lego. Modules can be created from a single or series of successive functions, comparably as in Lego structures made of several pieces (e.g., meant to build cars, houses, or roads). These modules (or atoms, tools) can be used standalone or combined to make simple to complex analytical workflows (e.g., data formatting or curation, running statistical models, or generating graphical elements for visualization). Doing so, the atomization approach may facilitate sharing or teaching analytical practices since beginners can easily understand the general organization of the analytical procedure by simply reading the list of steps in the analysis with a limited degree of complexity. Decoupling programming skills from analytical skills can make data processing more accessible to a wider audience. Indeed, once each elementary step is clearly identified and delimited along the atomization process, it is easier to grasp the whole analytical procedure and focus on the review of each step at a time or (re)use it. New workflows can further be generated by recombining existing, validated, or peer-reviewed elementary steps in innovative ways. This process can save time, increase confidence, and avoid potential programming mistakes, allowing greater focus on understanding the analytical workflow.

#### Generalization: what is it and why?

Generalization refers to the modification of an analytical procedure to make it applicable to many settings by removing specificities related to a particular data file or data format. This means trying to avoid hard-coding anything that is specific to the structure of the original dataset (e.g., number of years). Generalization aims to optimize the reusability at different times (e.g., regular result update) and enlarge the application of a given analysis to different input data files while keeping the initial analytical procedure fully reproducible, as pictured in the Graphical abstract 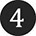. Generalizing an analytical step requires identifying key elements and invariant parameters from those that must be adaptable to allow for the analysis to be applied to specific characteristics of various datasets. These parameters must be implemented to be easily modified if needed. Generalization can be tricky because the higher the flexibility of an analytical step, the greater the risk of errors in its use. This is why generalization should be complemented by a clear statement and an implementation of red flags and warnings to prevent such events. As with atomization, generalization is primarily a conceptual way to build analytical procedures. It requires minor change of practices to reach a certain degree of generalization, avoiding additional effort later for reusability, reproducibility, and sharing.

#### Practical steps toward atomized and generalized coding

**Figure 1:**
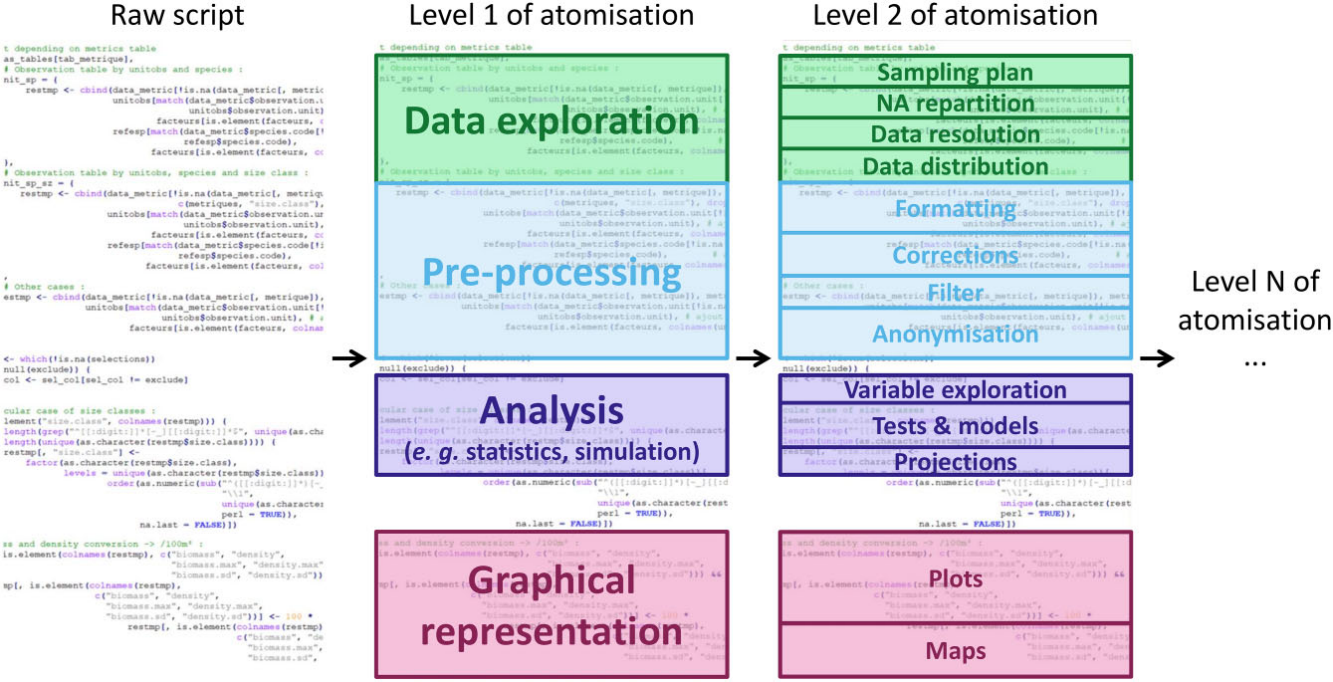
Illustration of the atomization of an existing code. The first level of atomization is delimitating the large sections of an analytical procedure that exist in almost all procedures. This first level is conveyed using same colors to the second level of atomization, where more detailed and specific analytical steps are illustrated in each section. The process of atomization can continue through a multitude of levels, ultimately leading to the maximally atomized procedure, which comprises a single function.

Breaking down codes into elementary steps to achieve atomization is not an intuitive task at first as it may target a single function or a more intricate set of several functions. There could be different degrees of atomization, depending on the grain required to decompose the analytical process (Fig. 1, Table 1). The application of general guidelines and best practices implies finding a balance between the most appropriate degree of atomization and generalization. This depends on the type of analytical procedure or the targeted audience (e.g., with different interests and programming skills). Attention to this balance is critical to ensure that the analytical procedures could be reused. For instance, a workflow in which each function would be considered a unique elementary step would optimize the flexibility but may likely add unnecessary complexity. At the other extreme, considering a whole analytical workflow as an elementary step may make it ready to use and simplify its application but would be too coarse and therefore limit flexibility by violating the principle of atomization.

**Table 1:** Example of atomization levels

Level 1—big shape	Level 2	Level 3
Data exploration	Sampling plan	Complete
		Balanced
	Missing values	Proportion
		Distribution
	Data granularity	Geographic resolution
		Temporal resolution
		Measure resolution
	Data distribution	Geographic coverage
		Temporal coverage
		Measures ranges
		Summaries
	…	…
Preprocessing	Formatting	Change file format
		Change general format
	Corrections	Remove special characters
		Remove low-trust observations
		Correct measures
	Filtering	Remove unwanted observations
	Anonymization	Anonymize names
		Anonymize localities
		Anonymize species
	…	…
Analysis	Variable exploration	PCA
		Collinearity
		Correlation
	Unimodal tests	Linear models
		χ²
		Student
	Statistical models	Generalized linear models
		Generalized additive models
		Random forest
	Model evaluation	Evaluation metrics (e.g., AIC, Jaccard)
		Validation methods
	Projections	Geographical projections
		Temporal projections
	…	…
Representation	Plot	Raw variables
		Modeled results
	Map	Observations
		Projections
	…	…

A few changes in code-writing habits can enhance the reusability of the analytical procedure by generating an easy-to-understand analytical procedure without investing much time. It is best to develop each elementary step directly in separate code files and to give details of the order in which elementary steps are used for each analytical workflow. To ensure reproducibility and traceability of the results, each computation of the analytical workflow should be associated with the details of the parameter settings and datasets used. From a practical point of view, a couple of recommendations could be made for coding elementary steps to facilitate generalization and ease the reuse. Once each elementary step is defined, we recommend all dependencies (e.g., software version, packages, libraries and their versions) to be set at the same place, at the start of the code, followed by modular parameters (e.g., input file location and name, column selection, modeling parameters, data specificities, output saving location). When the script of the elementary step is completed, modular parameters should be the only part of the code that may be modified in future reuse. Dependencies and subsequent computational tasks should be left untouched to ensure the integrity of the analysis and then reproducibility. In the end, it is best to add an open-source license to any analytical procedure shared publicly (e.g., MIT, GPL). It permits to clearly state the terms and conditions of diffusion, share, and reuse.

As such, atomization and generalization may overcome social or psychological barriers related to transparent sharing, related to securing ownership (e.g., DOI) and to embarrassment or fear during a peer-review process [29]. Indeed, as atomization and generalization notably permit higher readability of codes, it would be more straightforward for the writer or even trusted peers to verify and review the steps before submission.

Atomization and generalization are related and complementary concepts that may be applied from the earliest stages of the programming development. Indeed, atomization into adequate elementary steps is necessary to properly generalize an analytical procedure as it permits to enhance the modularity of the procedure and its capacity to be tailored to different data types.

### Entering a new dimension: the Galaxy-E initiative example

Developing open and properly atomized and generalized analytical procedures can already represent a significant step forward in terms of best practice. Galaxy is a good illustration of atomization and generalization with easier management of analytical workflows. The platform proposes many analytical tools that represent generalized and atomized elementary steps. These tools are modular and openly licensed, which permits building generalized workflows, as pictured in the Graphical abstract 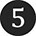.

Galaxy [23, 24] is a workflow-oriented web platform for analyzing data and sharing outputs. It allows scientists to share, develop, and use various datasets and data-processing tools (e.g., data formatting, statistical tests, graphic representations).

Galaxy enables good reproducibility for data exploration and analyses, helps compute intricate analyses on big data files, enables collaboration, and can support the teaching process. Galaxy-E is a Galaxy server dedicated to ecological analyses maintained by the European Galaxy team (supported by the German Federal Ministry of Education and Research and the German Network for Bioinformatics Infrastructure) and is available at https://ecology.usegalaxy.eu [49].

Galaxy-E is mostly aimed at scientists who process biodiversity data and already understand the general functioning of the analytical procedures they want to produce. The rationale for a user would be to create or reuse analytical workflows with high FAIRness in a collaborative and open source platform. It can be used for individual analyses as well as for collaborative projects. In some cases, if the analytical procedure is already clearly defined, it can be used by citizens or for teaching.

There are different Galaxy servers, at global, continental, and national levels (European and French levels, for example) but also according to the fields (e.g., biomedical, ecology, climate). The Galaxy-E initiative is hosted by European [49] and French [50] servers.

**Figure 2:**
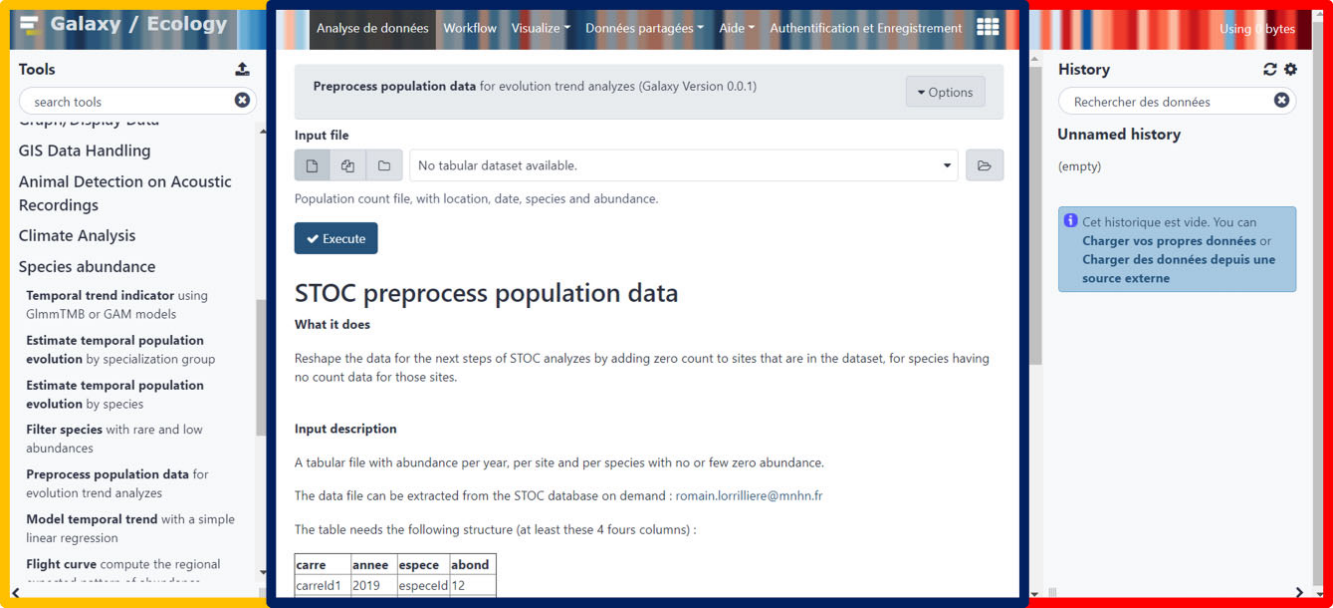
Galaxy-Ecology users’ interface [49, 50]. Yellow panel on the left: analysis tool list; blue panel in the middle: current tool interface; red panel on the right: Galaxy analysis history.

**Figure 3:**
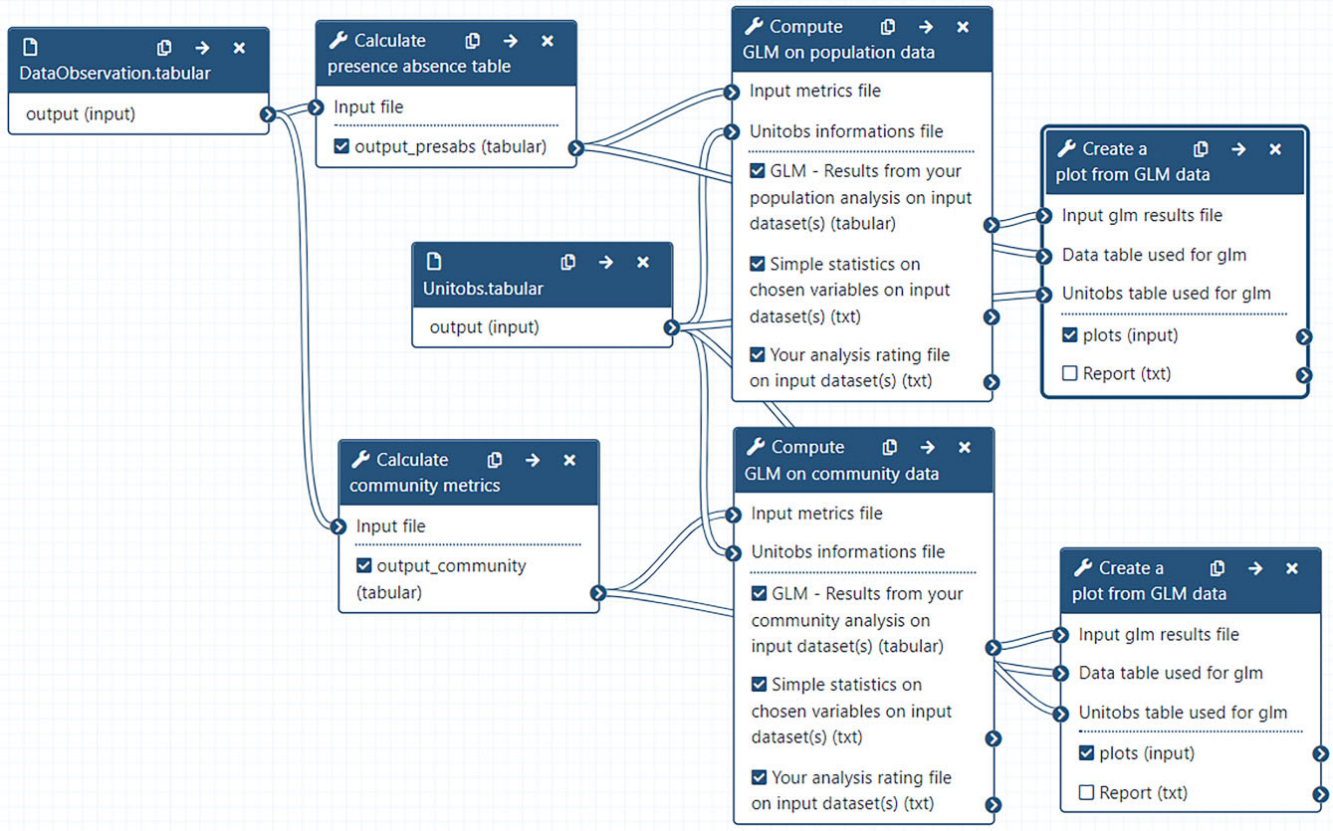
Representation of a Galaxy workflow in the editing interface of a Galaxy server. Each box represents an analysis tool, and the lines represent the flow of data through the tools. In relation with the atomization–generalization framework, each box (tool) corresponds to an atomized and generalized step with editable parameters, inputs, and outputs.

Datasets can be uploaded on a Galaxy server from a local device, an online server, or a database. Users can then access every available tool (Fig. 2, left panel) to modify, explore, and analyze their data. All tools used, parameters, and data (inputs and outputs) of the analysis are saved in a private “Galaxy history” (Fig. 2, right panel), documenting every step of the analytical procedure and recording the provenance of each output. From any history, the user can extract a workflow (Fig. 3) or directly share or publish the history itself. Workflows are reusable through WorkflowHub [51] or Dockstore [52] and exportable in CWL and RO-CRATE standards.

Any analytical procedure can be adapted on the platform, and Galaxy can be used through the whole data life cycle [53]. One can use off-the-shelf tools, workflows, and tutorials to design an analytical procedure or suggest, develop, and share new workflows and tutorials, 2 aspects that do not require coding skills.

As each Galaxy tool includes atomized and generalized elementary steps that can be articulated in a workflow, the Galaxy platform benefits from the same advantages as atomization and generalization and can help enhance best practice application (Table 2).

**Table 2:** Illustration of how the atomization–generalization framework and Galaxy implement and conform to best practice

		Atomized–generalized code	Galaxy
Reproducibility and transparency	Environment, software, and package versions	Can be indicated but possibly hard to manage Can also be set as an output of the analysis (e.g., session info) Packages written in each coded elementary step or using a versioning system such as Conda	Entirely packaged with a Conda package manager and BioContainers Possibility to store analytical procedures as containers for persistent execution
	Inputs and parameters	One must keep track of different parameterization and input settings at each computation	Automatically tracked and shareable with the “Galaxy history”
	Peer review	Organization of the analytical procedure reviewable by noncode developers Code developers might be able to detect errors as it is easier in shorter scripts Transparency over the development process achievable through Git	Reviewable “Galaxy history” and reexecutable workflow Continuous peer review of tools with open-source code Transparency over the development process through Git The workflows can be reviewed by the Intergalactic Workflow Commission (IWC) for best practices
	Output provenance	Can be tracked and reproduced in some cases	Tracked with the “Galaxy history” and reproducible with workflow
FAIR principles	Findable	If properly shared	Web-based solution Unified system for data and software citation and attribution Tools can be made available on several servers Tools can be linked to tool registries and annotated with different ontologies Annotated workflows findable on WorkflowHub [51] and Dockstore [52]
	Accessible	If properly shared	Free distribution of tools via the Galaxy ToolShed and workflows via WorkflowHub and Dockstore under an open-source license
	Interoperable	When properly generalized, different elementary steps should be useable in interaction with each other	Use different software, computational language, and library versions on a single platform with the Conda package management system Workflows exportable in JSON and shareable through several standards (e.g., Common Workflow Language [54] and Research Object Crate [55])
	Reusable	Generalized elementary steps are reusable and adaptable with different analytical procedure, parameterization, and/or inputs	Tools, histories, and workflows are reexecutable, reusable, and adaptable with different analytical procedure, parameterization, and/or inputs. Open-source code can be used outside of a Galaxy server
Technical and knowledge gaps	Understandability	The analytical procedure is clearer when properly atomized	Tools interface, workflow annotations, help sections, and tutorials are a valuable help
	Teaching opportunities	Learning the analytical procedure design separately from computing languages, giving structure to trainees Reusability of elementary steps for trainees	Experimenting with intricate analyses without computer code first Tutorials and videos from Galaxy Training Network [56] Galaxy community
	Computing capacity	Need for a computation cluster if large data or demanding algorithm	High-performance computing through an interface Bulk (meta)data manipulation
Collaboration and attribution	Analysis design and development	Achievable through collaborative code-editing applications	With anyone through a Galaxy server
	Citation	Easy reuse of openly shared elementary steps could lead to higher citation rates	Each tool, workflow, and tutorial are provided with a unique identifier for proper attribution and citation

The Galaxy platform emphasizes (i) accessibility of tools and data even without programming experience, (ii) reproducibility through the easy creation and reuse of analysis workflows, (iii) transparency through the open-source distribution of underlying codes, and (iv) community support.

For scientists, from a user’s point of view, it offers extensive computing power and a graphical interface to use analysis workflows, even without experience in software development. Web-based access allows easy sharing of analytical workflows between collaborators and with a broader audience. Galaxy supports tools in almost any computational language, including R and Python, 2 of the most used languages in ecology, with many packages dedicated to ecological and biodiversity-oriented analyses incorporated [57].

Anyone can use the tools on Galaxy and/or develop new tools and workflows to make them available to all by publishing them in the shared Galaxy ToolShed [58], which ensures that the tools and dependencies can be installed on any Galaxy servers. Any analytical procedure or workflow can be shared and enriched in parallel by several users, facilitating teamwork.

The platform is community-driven, which permits continuous peer review of the platform and the tools, workflows, and tutorials provided. Many tutorials are available on the Galaxy Training Network (GTN) [56], which is a valuable asset to the accessibility and reusability of tools and workflows [59, 60].

If enough researchers and experts start using and contributing to the platform, the number and content of available analytical procedures could expand at the same pace as latest analytical methodologies are integrated to research processes. If a different platform fits best and is more widely used by ecological and biodiversity scientific communities in the end, the work done on Galaxy will not be lost as tools are easily transposable to other interfaces (e.g., scripts directly usable with R, Python, etc., translation of workflows to other workflow engines).

Galaxy is ready to use and has proved its efficiency and suitability in other research fields, including genomics and climate science [61, 62]. Galaxy-Ecology has implemented workflows for biodiversity data exploration, environmental DNA processing, general population and community metrics and models, ecoregionalization, and normalized difference vegetation index (NDVI) computation with Sentinel-2 data, among others [63], with tutorials for several of them available on the GTN platform [64].

In addition to using existing tools, users may develop and upload entirely new tools and workflows to the Galaxy server in any computational language to make them accessible to all other users.

Galaxy is a participative platform, and several ways to participate in Galaxy exist depending on one’s skills, available time, and needs. Anyone can participate in the Galaxy-Ecology initiative by

– sharing datasets, histories, and workflows;
– giving feedback on servers, tools, and workflows;
– sharing tools and workflow ideas (eventually with code) through Git issues;
– asking for tool modifications through issues;
– modifying existing tools or proposing new tools through GitHub or GitLab;
– writing or contributing to a GTN tutorial on a specific functionality or a workflow on the GTN platform;
– creating learning pathways with a set of tutorials curated by community experts to form a coherent set of lessons around a topic and building knowledge [65]; and
– proposing training events and helping users in the utilization of a workflow and tutorial.

Analyses are rarely computed only once. Any analysis with a generalization potential is a suitable candidate to be Galaxy-fied. A methodological framework is presented in online supplementary material [66] at 3 levels depending on potential interests, computing language skills, and willingness to invest more or less time in the process: (i) “user” relying on existing Galaxy tools and workflows to analyze data (lower time investment), (ii) “developer” relying on an existing and validated analytical procedure to develop Galaxy tools and workflows (highest time investment), and (iii) “trainer” relying on existing Galaxy tools to share workflows and create training material (variable time investment).

### Discussion and limitations

Many best practices and recommendations exist for analytical procedures, data management, and computational code development. The levels of application of these best practices fall within a continuum offering a range of possibilities from the sole sharing of processed and interpreted results with a brief description of methods to an executable paper published within a container and emulated virtual machine [17, 67]. Situated somewhere in between the aforementioned extremes, the atomization–generalization framework and the utilization of the Galaxy platform might represent viable solutions offering a satisfactory level of best practices.

Atomization and generalization of computer codes can represent a relatively low investment strategy to attain certain levels of best practices such as transparency and reusability. It also carries advantages such as easier peer review, modularity of analytical procedures, and, consequently, time savings. Indeed, applying the framework is not sufficient to attain the highest levels of best practices. For reproducibility and transparency, the management of the environment, software, and package versions can be hard to maintain and record. For example, on a local computer, a comprehensive tracking of input, outputs, and codes requires meticulous management of folder structure in the environment. Additionally, noncode developers will be able to partially review the analytical procedure only if the workflow is clearly outlined in an adapted format (e.g., table, graphical representation). Accessibility and findability of the atomized and generalized analytical procedure are dependent on its proper sharing (e.g., persistent link, open repository).

Galaxy can represent an easier gateway toward higher levels of best practice as sharing a complete, detailed, and (re)executable analytical procedure is facilitated through provenance tracking and automatic metadata enrichment. In comparison, many scientific workflow management systems, such as Snakemake, Nextflow, or the R package Targets, operate from the command line. In ecology, numerous initiatives have tried to introduce such systems, starting with more user-friendly solutions—for example, the KNIME and Kepler systems with the CoESRA initiative (Collaborative Environment for Scholarly Research and Analysis) in Australia, Taverna with the BioVeL initiative (Biodiversity Virtual e-Laboratory) in Europe, or, very recently, the BON in a Box pipeline engine. These systems are more accessible to new users by offering a graphical interface while achieving high specificity [68–70]. However, good computer programming or scientific workflow management knowledge is still necessary to use these applications appropriately.

In comparison to the atomization–generalization framework, Galaxy can be rightfully seen as necessitating more time investment for scientists with programming experience as it requires learning to use a new platform. Additionally, more effort may be required on Galaxy when an additional analytical step needs to be developed, but the Galaxy community can be an efficient crutch on which hard-pressed scientists can rely. Indeed, one can ask for help on the implementation of tools whether one knows computing languages and can share their code or not.

## Conclusions

This article showcases a simple proposition to achieve best practices in analytical procedures with 2 plain guidelines: atomization and generalization. This straightforward framework represents a different manner to think and build analytical procedures; it does not require using a new technology or learning to use a new software. In terms of attaining higher levels of best practice, whether it is through the atomization–generalization framework, Galaxy, a combination of the two or otherwise, the optimal approach is to be determined by individuals depending on their interests, projects, and available resources. Relying on existing solutions as much as possible is, in our perspective, an efficient way to achieve a better understanding of best practices and their implications. Given the current environmental crisis, science has the major political and social responsibility to maintain good levels of transparency, reproducibility, and efficiency.

## Supplementary Material

giae122_Supplemental_File

giae122_GIGA-D-24-00463_Original_Submission

giae122_GIGA-D-24-00463_Revision_1

giae122_Response_to_Reviewer_Comments_Original_Submission

giae122_Reviewer_1_Report_Original_Submission Nick Isaac -- 10/31/2024

giae122_Reviewer_2_Report_Original_Submission Kari Norman -- 10/31/2024

## Data Availability

Data shared to test Galaxy training materials on the topics “Ecology” are available in Zenodo [83]. Test data are also associated with the Galaxy-Ecology tools GitHub repository available at [71] in the test data folder of each tool.

## Availability of Supporting Source Code and Requirements

Project name: Galaxy-Ecology tools

Project homepage: https://github.com/galaxyecology/tools-ecology [71]

Software Heritage PID: swh:1:dir:2d6d04c76c640f6796c6bb27abfd42c63028d4ca

Operating system(s): Platform independent, installation using the Galaxy Tool Shed, notably through the Ecology section: https://toolshed.g2.bx.psu.edu/repository/browse_repositories_in_category?id=b4146bb7fe9b8726&message=&status=done)

Programming language: R, Python, XSLT

License: MIT

This has also been archived in Software Heritage [72]

The Workflow Hub dedicated project is available at [63] with related workflows [73–81].

Galaxy training materials “Ecology” topics are available at [64] and associated workflows [, ].

## Abbreviations

GTN: Galaxy Training Network; NDVI: normalized difference vegetation index.
