# Supplementary material for: Guidance framework to apply best practices in ecological data analysis: lessons learned from building Galaxy-Ecology
Source: Gigascience. 2025 Feb 12;14:giae122. doi: 10.1093/gigascience/giae122 (PMC11816794; doi:10.1093/gigascience/giae122)
Supplement: giae122_Supplemental_File [file giae122_supplemental_file.docx]

**Table 2 -** Illustration of how the atomisation-generalisation framework and Galaxy implements and conforms to best practice.

|  |  | Atomised-generalised code | Galaxy |
| --- | --- | --- | --- |
| Reproducibility and transparency | Environment, software and package versions | Can be indicated but possibly hard to manage  Can also be set as an output of the analysis (*e.g.* session info)  Packages written in each coded elementary step or using a versioning system such as Conda | Entirely packaged with Conda package manager and BioContainers  Possibility to store analytical procedures as containers for persistent execution |
|  | Inputs and parameters | One must keep track of different parametrisation and input settings at each computation | Automatically tracked and shareable with the “Galaxy history” |
|  | Peer-review | Organisation of the analytical procedure reviewable by non-code developers  Code developers might be able to detect errors as it is easier in shorter scripts  Transparency over the development process achievable through Git | Reviewable “Galaxy history” and re-executable workflow  Continuous peer-reviewed of tools with open-source code  Transparency over the development process through Git  The workflows can be reviewed by the Intergalactic Workflow Commission (IWC) for best practices |
|  | Output provenance | Can be tracked and reproduced in some cases | Tracked with the “Galaxy history” and reproducible with workflow |
| FAIR principles | Findable | If properly shared | Web-based solution  Unified system for data and software citation and attribution  Tools can be made available on several servers  Tools can be linked to tools registries and annotated with different ontologies  Annotated workflows findable on WorkflowHub [51] and Dockstore [52] |
|  | Accessible | If properly shared | Free distribution of tools via the Galaxy ToolShed and workflows via WorkflowHub and Dockstore under an open-source licence |
|  | Interoperable | When properly generalised, different elementary steps should be useable in interaction with each other | Use different software, computational language and library versions on a single platform with the Conda package management system  Workflows exportable in JSON and shareable through several standards (*e.g.* Common Workflow Language [54] and Research Object Crate [55]) |
|  | Reusable | Generalised elementary steps are reusable and adaptable with different analytical procedure, parametrisation and/or inputs | Tools, histories and workflows are re-executable, reusable and adaptable with different analytical procedure, parametrisation and/or inputs. Open-source code can be used outside of a Galaxy server |
| Technical and knowledge gaps | Understandability | The analytical procedure is clearer when properly atomised | Tools interface, workflow annotations, help sections and tutorials are a valuable help |
|  | Teaching opportunities | Learning the analytical procedure design separately from computing languages, giving structure to trainees  Reusability of elementary steps for trainees | Experimenting with intricate analyses without computer code first  Tutorials and videos from Galaxy Training Network [56]  Galaxy community |
|  | Computing capacity | Need for a computation cluster if large data or demanding algorithm | HPC (High Performance Computing) through an interface  Bulk (meta)data manipulation |
| Collaboration and attribution | Analysis design and development | Achievable through collaborative code-editing applications | With anyone through a Galaxy server |
|  | Citation | Easy reuse of openly shared elementary steps could lead to higher citation rates | Each tool, workflow, and tutorial are provided with a unique identifier for proper attribution and citation |
