# Supplementary material for: Guidance framework to apply best practices in ecological data analysis: lessons learned from building Galaxy-Ecology
Source: Gigascience. 2025 Feb 12;14:giae122. doi: 10.1093/gigascience/giae122 (PMC11816794; doi:10.1093/gigascience/giae122)

GigaScience

Guidance framework to apply best practices in ecological data analysis: Lessons learned from building Galaxy-Ecology

--Manuscript Draft--

**Manuscript Number:**	GIGA-D-24-00463R1
**Full Title:**	Guidance framework to apply best practices in ecological data analysis: Lessons learned from building Galaxy-Ecology
**Article Type:**	Review
**Funding Information:**	

Erasmus+ (2020-1-NL01-KA203-064717)	Not applicable
Agence Nationale de la Recherche (65 Million d'Observateurs)	Not applicable
Agence Nationale de la Recherche (IA-Biodiv)	Not applicable
Fonds national pour la science ouverte (OpenMetaPaper)	Not applicable
Horizon 2020 (EOSC-Pillar)	Not applicable
Horizon 2020 (GAPARS)	Not applicable
HORIZON EUROPE Framework Programme (FAIRE EASE)	Not applicable
HORIZON EUROPE Framework Programme (GO FAIR BiodiFAIRse Implementation Network)	Not applicable
Blue Nature Alliance	Not applicable
Antarctic and Southern Ocean Coalition	Not applicable
French Ministry of Higher Education and Research	Not applicable

Abstract:

Numerous conceptual frameworks exist for best practices in research data and analysis (e.g. Open Science and FAIR principles). In practice, there is a need for further progress to improve transparency, reproducibility, and confidence in ecology. Here, we propose a practical and operational framework for researchers and experts in ecology to achieve best practices for building analytical procedures from individual research projects to production-level analytical pipelines. We introduce the concept of atomisation to identify analytical steps which support generalisation by allowing us to go beyond single analyses.

The term atomisation is employed to convey the idea of single analytical steps as “atoms” composing an analytical procedure. When generalised, “atoms” can be used in more than a single case analysis. These guidelines were established during the development of the Galaxy-Ecology initiative, a web platform dedicated to data analysis in ecology. Galaxy- Ecology allows us to demonstrate a way to reach higher levels of reproducibility in ecological sciences by increasing the accessibility and reusability of analytical workflows once atomised and generalised.

**Corresponding Author:**	Coline Royaux<br>Sorbonne Université Campus Pierre et Marie Curie: Sorbonne Universite Campus Pierre et Marie Curie<br>Concarneau, FRANCE
**Corresponding Author Secondary Information:**	
**Corresponding Author's Institution:**	Sorbonne Université Campus Pierre et Marie Curie: Sorbonne Universite Campus Pierre et Marie Curie
**Corresponding Author's Secondary Institution:**	

Powered by Editorial Manager® and ProduXion Manager® from Aries Systems Corporation

**First Author:**	Coline Royaux
**First Author Secondary Information:**	
**Order of Authors:**	Coline Royaux
	Jean-Baptiste Mihoub
	Marie Jossé
	Dominique Pelletier
	Olivier Norvez
	Yves Reecht
	Anne Fouilloux
	Helena Rasche
	Saskia Hiltemann
	Bérénice Batut
	Eleaume Marc
	Pauline Seguineau
	Guillaume Massé
	Alan Amossé
	Claire Bissery
	Romain Lorrilliere
	Alexis Martin
	Yves Bas
	Thimothée Virgoulay
	Valentin Chambon
	Elie Arnaud
	Elisa Michon
	Clara Urfer
	Eloïse Trigodet
	Marie Delannoy
	Grégoire Loïs
	Romain Julliard
	Björn Grüning
	Yvan Le Bras
**Order of Authors Secondary Information:**	
**Response to Reviewers:**	Comments from Nicole Nogoy<br><br>- Please list the following for the Galaxy-E platform:<br>Project name: e.g. My bioinformatics project<br>Project home page: e.g. https://github.com/ISA-tools<br>Operating system(s): e.g. Platform independent<br>Programming language: e.g. Java<br>Other requirements: e.g. Java 1.3.1 or higher, Tomcat 4.0 or higher<br>License: e.g. GNU GPL, FreeBSD etc.<br>RRID: if applicable, e.g. RRID: SCR_014986<br>Also; cite the Workflow Hub DOI.<br>- Response : It has been added.

	- please also include information on supporting data used to demonstrate the Galaxy-E platform here listing accessions.<br>- Response : It has been added.<br>- Please add abbreviations here used more than once in alphabetical order.<br>- Response : There are no abbreviations in the text if we are not mistaken.
**Additional Information:**	
**Question**	**Response**
Are you submitting this manuscript to a special series or article collection?	No
**Experimental design and statistics**<br><br>Full details of the experimental design and statistical methods used should be given in the Methods section, as detailed in our Minimum Standards Reporting Checklist. Information essential to interpreting the data presented should be made available in the figure legends.<br><br>Have you included all the information requested in your manuscript?	Yes
**Resources**<br><br>A description of all resources used, including antibodies, cell lines, animals and software tools, with enough information to allow them to be uniquely identified, should be included in the Methods section. Authors are strongly encouraged to cite Research Resource Identifiers (RRIDs) for antibodies, model organisms and tools, where possible.<br><br>Have you included the information requested as detailed in our Minimum Standards Reporting Checklist?	Yes
**Availability of data and materials**<br><br>All datasets and code on which the conclusions of the paper rely must be either included in your submission or deposited in publicly available repositories (where available and ethically appropriate), referencing such data using	Yes

Powered by Editorial Manager® and ProduXion Manager® from Aries Systems Corporation

a unique identifier in the references and in the “Availability of Data and Materials” section of your manuscript.<br><br>Have you have met the above requirement as detailed in our Minimum Standards Reporting Checklist?	
GigaScience has policies and guidelines in place for the use of generative AI-writing tools such as ChatGPT. If you have used such writing tools to assist with writing the manuscript this must be declared and cited in the text. Authors should not list AI-writing tools and other AI-assisted technologies as an author or co-author and should acknowledge that they are fully responsible for text generated or refined by AI-writing tools.<p><br><br>A summary of use (particularly in the introduction or among methods) needs to be included at the end of the paper, and the outputs should also be included as a supplementary file hosted in GigaDB or other open repositories. Please read our guidelines for more information. <p><br><br>By submitting to GigaScience, you are aware of the journal's AI-writing tools policy, and if you have declared use of such tools below, you have acknowledged this where appropriate in your manuscript and have made a summary of use and outputs available. <p><br><b>AI-assisted writing tools have been used in the preparation of this manuscript?	No

Powered by Editorial Manager® and ProduXion Manager® from Aries Systems Corporation

Manuscript
Click here to access/download;Manuscript;MEP_GS_revGS NN edits.docx

Guidance framework to apply best practices in ecological data analysis: Lessons learned from building Galaxy-Ecology

PCI recommendation : https://doi.org/10.24072/pci.ecology.100694

Royaux Coline[1,2*]: coline.royaux@mnhn.fr; royaux.c@gmail.com

Mihoub Jean-Baptiste[3] : jean-baptiste.mihoub@mnhn.fr

Jossé Marie[4]: marie.josse@ifremer.fr

Pelletier Dominique[5]: dominique.pelletier@ifremer.fr

Norvez Olivier[6]: olivier.norvez@mnhn.fr

Reecht Yves[7,8]: yves.reecht@hi.no

Fouilloux Anne[9]: annef@simula.no

Rasche Helena[10]: helenarasche@gmail.com

Hiltemann Saskia[11]: saskiahiltemann@gmail.com

Batut Bérénice[12,13]: berenice.batut@france-bioinformatique.fr

Eléaume Marc[14,15]: marc.eleaume@mnhn.fr

Seguineau Pauline[14,15]: pauline.seguineau@mnhn.fr

Massé Guillaume[16]: guillaume.masse@mnhn.fr

Amossé Alan[17]: alan.amosse@gmail.com

Bissery Claire[8,18]: cbissery@wanadoo.fr

Lorrilliere Romain[3]: romain.lorrilliere@mnhn.fr

Martin Alexis[19]: alexis.martin@mnhn.fr

Bas Yves[3,20]: yves.bas@mnhn.fr

Virgoulay Thimothée[21,22]: Tim.Virgoulay@nrm.se

Chambon Valentin[17]: v.chambon.menand@gmail.com

Arnaud Elie[2]: elie.jjm.arnaud@gmail.com

Michon Elisa[23]: elisa.michon@uqar.ca

Urfer Clara[2,24]: claraurf@hotmail.com

Trigodet Eloïse[21,24]

Delannoy Marie[3]: my.delannoy@gmail.com

Loïs Gregoire[3]

Julliard Romain[3]: romain.julliard@mnhn.fr

Grüning Björn[25]: bjoern.gruening@gmail.com

The Galaxy-E community

Le Bras Yvan[2]: yvan.le-bras@mnhn.fr

[1] UMR8067 Biologie des Organismes et Ecosystèmes Aquatiques (BOREA, MNHN-CNRS-SU-IRD-UCN-UA), Sorbonne Université, Station Marine de Concarneau - Concarneau, France

[2] Pôle national de données de biodiversité, UAR2006 PatriNat (OFB-MNHN-CNRS-IRD), Muséum National d'Histoire Naturelle, Station Marine de Concarneau - Concarneau, France

[3] Centre d'Écologie et des Sciences de la Conservation (UMR7204 CESCO, MNHN-CNRS-SU), Muséum National d'Histoire Naturelle, Sorbonne Université, Centre National de la Recherche Scientifique - Paris, France

[4] Data Terra, Centre National de la Recherche Scientifique - Brest, France

[5] UMR DECOD (Ifremer-Agrocampus Ouest-INRAE) - Lorient, France

[6] Pôle National de Données de Biodiversité, UAR2006 PatriNat (OFB-MNHN-CNRS-IRD), Fondation pour la Recherche sur la Biodiversité, Muséum national d'Histoire naturelle - Paris, France

[7] Institute of Marine Research - Bergen, Norway

[8] Institut français de recherche pour l'exploitation de la mer (Ifremer) – Brest, France

[9] Simula Research Laboratory - Oslo, Norway

[10] Department of Pathology and Clinical Bioinformatics, Erasmus Medical Center - Rotterdam, The Netherlands

[11] Institute of Pharmaceutical Sciences, Faculty of Chemistry and Pharmacy, University of Freiburg - Freiburg, Germany

[12] Institut Français de Bioinformatique, CNRS UAR3601 - Évry, France

[13] Mésocentre, Clermont-Auvergne, Université Clermont Auvergne - Clermont-Ferrand, France

[14] Institut de Systématique Evolution, Biodiversité (UMR7205 ISYEB, MNHN-CNRS-SU-EPHE), Département Origines et Évolution, Muséum national d'Histoire naturelle - Paris, France

[15] Institut de Systématique Evolution, Biodiversité (UMR7205 ISYEB, MNHN-CNRS-SU-EPHE), Département Origines et Évolution, Station Marine de Concarneau - Concarneau, France

[16] UMR LOCEAN (CNRS-SU-IRD-MNHN), Centre National de la Recherche Scientifique, Station Marine de Concarneau - Concarneau, France

[17] Muséum National d'Histoire Naturelle, Station Marine de Concarneau - Concarneau, France

[18] Université Claude Bernard Lyon 1 - Lyon, France

[19] UMR8067 Biologie des Organismes et Ecosystèmes Aquatiques (BOREA, MNHN-CNRS-SU-IRD-UCN-UA), Muséum national d'Histoire naturelle - Paris, France

[20] UAR2006 PatriNat (OFB-MNHN-CNRS-IRD), Muséum national d'Histoire naturelle - Paris, France

[21] Centre d'Écologie et des Sciences de la Conservation (UMR7204 CESCO, MNHN-CNRS-SU), Muséum National d'Histoire Naturelle - Concarneau, France

[22] Université de Montpellier - Montpellier, France

[23] Institut des Sciences de la Mer de Rimouski, Université du Québec à Rimouski - Rimouski, Québec, Canada

[24] Université de Bretagne Occidentale - Brest, France

[25] Bioinformatics Group, Department of Computer Science, Albert-Ludwigs-University Freiburg - Freiburg, Germany

*Corresponding author

ABSTRACT

Numerous conceptual frameworks exist for best practices in research data and analysis (*e.g.* Open Science and FAIR principles). In practice, there is a need for further progress to improve transparency, reproducibility, and confidence in ecology. Here, we propose a practical and operational framework for researchers and experts in ecology to achieve best practices for building analytical procedures from individual research projects to production-level analytical pipelines. We introduce the concept of atomisation to identify analytical steps which support generalisation by allowing us to go beyond single analyses. The term atomisation is employed to convey the idea of single analytical steps as "atoms" composing an analytical procedure. When generalised, "atoms" can be used in more than a single case analysis. These guidelines were established during the development of the Galaxy-Ecology initiative, a web platform dedicated to data analysis in ecology. Galaxy-Ecology allows us to demonstrate a way to reach higher levels of reproducibility in ecological sciences by increasing the accessibility and reusability of analytical workflows once atomised and generalised.

Graphical abstract – Levels of attainable best practices through the atomisation – generalisation framework

Keywords: Biodiversity; Reproducible analyses; Galaxy; Best practices; Atomisation; Generalisation; Workflows; Ecoinformatics

Background

Ecology's Reproducibility Crisis

Research in ecology is increasingly shaped by the availability of novel analytical solutions and statistical tools. Given the ever-growing amount of data available, much attention is often given to the thought process behind statistical analyses to handle different data distributions, pseudo-replication, and sampling biases for instance [1 – 3]. Despite the high-quality standards required by the scientific community from data access to analysis, the level of complexity of ecological systems makes results difficult to reproduce. The ongoing "reproducibility crisis" has also led researchers to pay closer attention to the quality of analyses to increase confidence in their studies and conclusions [4, 5]. Reproducibility (*i.e.* different teams and experimental setups obtaining similar results) [6] is one of the main criteria for evaluating robust science and reliable conclusions. The term "reproducibility" is a relative concept and has known various definitions depending on field and context. Reproducibility of analyses ("computational reproducibility") is defined by Cohen-Boulakia *et al.* [7] as the ability of distinct analyses to reach to the same conclusion.

In the current context of the global biodiversity crisis, the scientific community needs to use all available data and provide as robust as possible evidence regarding the state and dynamic of ecological systems, from genetic to ecosystem. At the same time, using analytical tools to provide robust evidence can be complex and may require advanced skills that are not widely available across the scientific community [2]. Therefore, operational solutions and methodological guidelines can allow analytical workflows to be more accessible without degrading the scientific quality of analyses, and thus, promote efficient and broad deployment of best practices.

Is the ecology community failing to meet best practices?

The first step towards reproducibility is knowing current best practices and recommendations. Among them, the FAIR principles [8], for which the availability of the data and the code used for each published result is an essential criterion, may be key for appropriate management through the data life cycle [9]. The FAIR principles (see also CARE principles [10]) are considered as a founding framework to share data along four important elements: "Findable" for humans and machines; "Accessible" with a detailed access procedure; "Interoperable" for interaction with other data or applications; "Reusable" in an identical or different context. In addition to these principles, propositions have been delimited within several thematic communities in ecology to evaluate and enhance best practices application, notably the Species Distribution Modelling communities [11, 12]. Although data accessibility has been substantially improved in ecology during the past decade, sharing analytical scripts and codes remain largely marginal [13 - 16]. However, even if sharing code is necessary to achieve good computational reproducibility, it is insufficient. Therefore, the utilisation of computational workflows has been suggested as a solution for improving computational reproducibility [7, 17] through software such as Snakemake [18 - 20], Nextflow [21, 22], or Galaxy [23, 24]. A workflow is generally defined as a sequence of distinct computational tasks for a particular objective [25]. As such, a workflow represents the backbone of a single specific analysis. Throughout the analytical procedure, a typical workflow starts with raw data, which can be extracted from several databases or data files and processed through a series of analytical steps. The products resulting from these analytical steps (*i.e.* the outputs of the computational workflow) can be data files, graphic representations and any associated metrics.

When properly designed, a certain level of reproducibility can be easily achieved since workflow languages naturally capture the following four key elements [7]:

- the specificities of the workflow, the analysis steps and associated tools;

– the workflow entries, datasets and parameters;
– the environment and context of the use of the workflow;
– the results obtained and the outputs of the workflow.

In the original publication of Wilkinson *et al.* [8], the focus of FAIR principles was mainly on observational data. However, the principles can be applied to software and computational workflows [25, 26]. For instance, a code shared as supplementary material of a non-open access publication could be considered as "Interoperable" but is not easily "Findable", "Accessible", or "Reusable". In contrast, a large block of code consisting of several hundred lines, from data pre-processing to final results and graphics as pictured in the Graphical abstract ❶, may require efforts to understand and adapt to other kinds of data ("non-reusable"), mainly if annotations or comments are limited. Similarly, an analytical procedure shared without indicating the versions of hardware, software, and packages has a low chance of producing identical outputs, making it less reproducible. These issues may harm the scientific community by preventing fully transparent communication among users about knowledge production and practice comparison. They can also be detrimental to individual authors, when they need to update or run new analyses.

Impact on Ecology Research

The efficiency of the scientific process is greatly affected by the lack of computational reproducibility and FAIRness of analytical procedures. The adoption of FAIR practices was estimated to save 10.2 billion € per year in Europe [27 - 29]. Moreover, consistent application of reproducibility and FAIR principles will improve trust in research studies and scientific reports [30 - 32].

The widespread use of computational languages to process large-scale data and analyse complex systems has been a major advance in studying the ecosphere at any spatio-temporal scale [33, 34]. However, the ever-growing technical and programming skills

required to take advantage of such computational solutions by the scientific community raise new challenges [35 - 37]. The use of increasingly complex analytical solutions, paired with different approaches or programming languages, creates barriers to uptake and challenges for peer-review. Indeed, many ecologists have acquired their programming skills through self-study or through courses that combine instruction in statistics and ecological principles with an introduction to programming. This learning process does not inherently compromise the quality of the analyses and results; however, it may lead to inappropriate coding habits. As a response to this situation, adequate training was identified by life science researchers [38 - 40], as it would help involve more people in the understanding of current analytical solutions and benefit to scientific cooperation [41, 42]. Research is typically structured through a highly competitive organisation, with a potentially detrimental effect on scientific knowledge [43]. Instead, fostering collaboration and collective intelligence by promoting transparent sharing of analytical procedures, would offer more persistent and robust ways to achieve actionable science [44]. Such efforts would be of paramount importance in environmental sciences and the conservation of biodiversity by providing governance and guiding actions with increasingly robust evidence [45].

Are there simple and ready-to-use solutions?

In this article, we aim to promote the reuse of existing concepts and solutions as pillars toward better practices for ecological analyses by providing a streamlined framework. We believe the atomisation-generalisation framework presented in the second part of this article represents an operational and actionable path for researchers and experts to attain levels of best practices (*e.g.* reproducibility, FAIR, open science, R compendium) [46] with no more investment than they are able or willing to provide [47]. Atomisation is used to refer to the identification of distinct analytical steps each constituting an analytical

procedure. It is a non-standard term introduced in this article to convey the idea of analytical "atoms". As for atom particles that etymologically correspond to "indivisible" but are composed of subatomic particles, an analytical atom represents a single analytical step composed of several functions. Generalisation involves the alteration of an analytical step to enlarge its applicability in diverse contexts and for diverse purposes. Therefore, generalisation cannot be efficiently achieved without prior atomisation.

Atomisation and Generalisation are central organising principles in the design of the Galaxy-Ecology (Galaxy-E) initiative (see section III). Galaxy-E is a demonstration platform for applying best practices such as the FAIR principles and computational reproducibility for analytical procedures in ecology. Hence, this review article is partly Galaxy-oriented, not to present the platform as a prescriptive solution but to give an operational example of the best practices it helps to achieve.

Main text

Guidelines for best practices

Atomisation: what is it and why?

Atomisation refers to dividing an analytical procedure into several specific steps ("atoms"; Graphical abstract ❷) generating a suite of elementary analytical steps as pictured in the Graphical abstract ❸. For instance, in a maximally-atomised workflow, each small step would be conducted by its own bespoke function. Breaking down the analytical process into atoms functioning as building blocks allows for better understanding, modularity, and visibility of the analytical flow. It permits making it more accessible to a broader audience or facilitating the peer-review process. Indeed, an extended one-block code that imports raw data, makes pre-processing steps (*e.g.* filter, formatting), conducts analyses (*e.g.* distribution study, modelling), and performs final representations of results (*e.g.* maps,

plots) can be challenging to understand and reuse by others or even the same person after some time.

McIntire *et al.* [48] described the PERFICT approach (Prediction, Evaluation, Reusability, Free access, Interoperability, Continuous workflows, and routine Tests) to set a new foundation for models in predictive ecology. This can be applied more generally to the analytical procedure in ecology and biodiversity. In their article, McIntire and collaborators make an analogy between code development and Lego® construction, similar to our definition of atomisation. Functions are a workflow's most fundamental analytical steps and can be seen as modular pieces, alike single pieces of Lego®. Modules can be created from a single or series of successive functions comparably as in Lego® structures made of several pieces (*e.g.* meant to build cars, houses, or road). These modules (or atoms, tools) can be used as standalone or combined to make simple to complex analytical workflows (*e.g.* data formatting or curation, running statistical models, or generating graphical elements for visualisation). Doing so, the atomisation approach may facilitate sharing or teaching analytical practices since beginners can easily understand the general organisation of the analytical procedure by simply reading the list of steps in the analysis with a limited degree of complexity. Decoupling programming skills from analytical skills can make data processing more accessible to a wider audience. Indeed, once each elementary step is clearly identified and delimited along the atomisation process, it is easier to grasp the whole analytical procedure and focus on the review of each step at a time or (re)use it. New workflows can further be generated by recombining existing, validated or peer-reviewed elementary steps in innovative ways. This process can save time, increase confidence, and avoid potential programming mistakes, allowing greater focus on understanding the analytical workflow.

Generalisation: what is it and why?

Generalisation refers to the modification of an analytical procedure to make it applicable to many settings, by removing specificities related to a particular data file or data format. This means trying to avoid hard-coding anything that is specific to the structure of the original dataset (e.g. number of years). Generalisation aims to optimise the reusability at different times (*e.g.* regular result update), enlarge the application of a given analysis to different input data files while keeping the initial analytical procedure fully reproducible as pictured in the Graphical abstract ❹. Generalising an analytical step requires identifying key elements and invariant parameters from those that must be adaptable to allow for the analysis to be applied to specific characteristics of various datasets. These parameters must be implemented to be easily modified if needed. Generalisation can be tricky because the higher the flexibility of an analytical step, the greater the risk of errors in its use. This is why generalisation should be complemented by clear statement and an implementation of red flags and warnings to prevent such events. As with atomisation, generalisation is primarily a conceptual way to build analytical procedures. It requires minor change of practices to reach certain degree of generalisation, avoiding additional effort later for reusability, reproducibility, and share.

Practical steps towards atomised and generalised coding

Breaking down codes into elementary steps to achieve atomisation is not an intuitive task at first as it may target a single function or a more intricate set of several functions. There could be different degrees of atomisation, depending on the grain required to decompose the analytical process (fig. 1; tab. 1). The application of general guidelines and best practices implies finding a balance between the most appropriate degree of atomisation and generalisation. This depends on the type of analytical procedure or the targeted audience (*e.g.* with different interests and programming skills). Attention to this balance is critical to

ensure that the analytical procedures could be reused. For instance, a workflow in which each function would be considered as a unique elementary step would optimise the flexibility but may likely add unnecessary complexity. At the other extreme, considering a whole analytical workflow as an elementary step may make it ready-to-use and simplify its application, but would be too coarse and therefore limit flexibility by violating the principle of atomisation.

Figure 1 - Illustration of the atomisation of an existing code. The first level of atomisation is delimitating the large sections of an analytical procedure that exist in almost all procedures. This first level is conveyed using same colours to the second level of atomisation where more detailed and specific analytical steps are illustrated in each section. The process of atomisation can continue through a multitude of levels, ultimately leading to the maximally atomised procedure, which is comprised of a single function.

Table 1 - Example of atomisation levels

Level 1 - big shape	Level 2	Level 3
Data exploration	Sampling plan	Complete
		Balanced
	Missing values	Proportion
		Distribution
	Data granularity	Geographic resolution
		Temporal resolution
		Measure resolution
	Data distribution	Geographic coverage
		Temporal coverage
		Measures ranges
		Summaries
	…	…
Pre-processing	Formatting	Change file format
		Change general format
	Corrections	Remove special characters
		Remove low trust observations
		Correct measures
	Filtering	Remove unwanted observations
	Anonymisation	Anonymise names
		Anonymise localities

		Anonymise species
	…	…
Analysis	Variable exploration	PCA
		Collinearity
		Correlation
	Unimodal tests	Linear Models
		χ^2
		Student
	Statistical models	Generalised Linear Models
		Generalised Additive Models
		Random Forest
	Models Evaluation	Evaluation metrics (*e.g.* AIC, Jaccard)
		Validation methods
	Projections	Geographical projections
		Temporal projections
	…	…
Representation	Plot	Raw variables
		Modelled results
	Map	Observations
		Projections
	…	…

A few changes in code-writing habits can enhance the reusability of the analytical procedure by generating easy-to-understand analytical procedure without investing much time. It is best to develop each elementary step directly in separate code files and to give details of the order in which elementary steps are used for each analytical workflow. To ensure reproducibility and traceability of the results, each computation of the analytical workflow should be associated with the details of the parameters settings and datasets used. From a practical point of view, a couple of recommendations could be made for coding elementary steps to facilitate generalisation and ease the reuse. Once each elementary step is defined, we recommend all dependencies (*e.g.* software version, packages, libraries and their versions) to be set at the same place, at the start of the code, followed by modular parameters (*e.g.* input file location and name, column selection, modelling parameters, data specificities, output saving location). When the script of the elementary step is completed, modular parameters should be the only part of the code that may be modified in future reuse. Dependencies and subsequent computational tasks should be left untouched to ensure the integrity of the analysis and then, reproducibility. In the end, it is best to add an

open-source license to any analytical procedure shared publicly (*e.g.* MIT, GPL). It permits to clearly state the terms and conditions of diffusion, share and reuse.

As such, atomisation and generalisation may overcome social or psychological barriers related to transparent sharing, either related to securing ownership (*e.g.* DOI) and to embarrassment or fear during a peer-review process [29]. Indeed, as atomisation and generalisation notably permit higher readability of codes, it would be more straightforward for the writer or even trusted peers to verify and review the steps before submission.

Atomisation and generalisation are related and complementary concepts that may be applied from the earliest stages of the programming development. Indeed, atomisation into adequate elementary steps is necessary to properly generalise an analytical procedure as it permits to enhance the modularity of the procedure and its capacity to be tailored to different data types.

Entering a new dimension: the Galaxy-E initiative example

Developing open and properly atomised and generalised analytical procedures can already represent a significant step forward in terms of best practice. Galaxy is a good illustration of atomisation and generalisation with easier management of analytical workflows. The platform proposes many analytical tools that represent generalised and atomised elementary steps. These tools are modular and openly licensed, which permits to build generalised workflows as pictured in the Graphical abstract ❺.

Galaxy [23, 24] is a workflow-oriented web platform for analysing data and sharing outputs. It allows scientists to share, develop, and use various datasets and data processing tools (e.g. data formatting, statistical tests, graphic representations).

Galaxy enables good reproducibility for data exploration and analyses, helps compute intricate analyses on big data files, enables collaboration, and can support the teaching process. Galaxy-E is a Galaxy server dedicated to ecological analyses maintained by the

European Galaxy team (supported by the German Federal Ministry of Education and Research and the German Network for Bioinformatics Infrastructure), and is available at https://ecology.usegalaxy.eu [49].

Galaxy-E is mostly aimed at scientists that process biodiversity data and already understand the general functioning of the analytical procedures they want to produce. The rationale for a user would be to create or reuse analytical workflows with high FAIRness in a collaborative and open source platform. It can be used for individual analyses as well as for collaborative projects. In some cases, if the analytical procedure is already clearly defined, it can be used by citizens or for teaching.

There are different Galaxy servers, at global, continental, and national levels (European and French levels for example), but also according to the fields (*e.g.*, biomedical, ecology, climate). The Galaxy-E initiative is hosted by European [49] and French [50] servers. Datasets can be uploaded on a Galaxy server from a local device, an online server, or a database. Users can then access every available tool (fig. 2, left panel) to modify, explore, and analyse their data. All tools used, parameters, and data (inputs and outputs) of the analysis are saved in a private "Galaxy history" (fig. 2, right panel), documenting every step of the analytical procedure and recording the provenance of each output. From any history, the user can extract a workflow (fig. 3) or directly share or publish the history itself. Workflows are reusable through WorkflowHub [51] or Dockstore [52] and exportable in CWL and RO-CRATE standards.

Figure 2 - Galaxy-Ecology users' interface [49, 50]. Yellow panel on the left: analysis tool list; blue panel in the middle: current tool interface; red panel on the right: Galaxy analysis history

Figure 3 - Representation of a Galaxy workflow in the editing interface of a Galaxy server. Each box represents an analysis tool, and the lines represent the flow of data through the tools. In relation with the atomisation-generalisation

framework, each box (tool) corresponds to an atomised and generalised step with editable parameters, inputs and outputs.

Any analytical procedure can be adapted on the platform and Galaxy can be used through the whole data life cycle [53]. One can use off-the-shelf tools, workflows, and tutorials to design an analytical procedure, or suggest, develop, and share new workflows and tutorials, two aspects that do not require coding skills.

As each Galaxy tools are atomised and generalised elementary steps that can be articulated in a workflow, the Galaxy platform benefits from the same advantages as atomisation and generalisation and can help enhancing best practice application (tab. 2).

Table 2 - Illustration of how the atomisation-generalisation framework and Galaxy implements and conforms to best practice. [Large table see additional file].

The Galaxy platform emphasises (i) accessibility of tools and data even without programming experience, (ii) reproducibility through the easy creation and reuse of analysis workflows, (iii) transparency through the open-source distribution of underlying codes; and (iv) community support.

For scientists, from a user's point of view, it offers extensive computing power and a graphical interface to use analysis workflows, even without experience in software development. Web-based access allows easy sharing of analytical workflows between collaborators and with a broader audience. Galaxy supports tools in almost any computational language, including R and Python, two of the most used languages in ecology, with many packages dedicated to ecological and biodiversity-oriented analyses incorporated [57].

Anyone can use the tools on Galaxy and/or develop new tools and workflows to make them available to all by publishing them in the shared Galaxy ToolShed [58] which ensures that the tools and dependencies can be installed on any Galaxy servers. Any analytical procedure or workflow can be shared and enriched in parallel by several users, facilitating teamwork. The platform is community-driven which permits continuous peer review of the platform and of the tools, workflows and tutorials provided. Many tutorials are available on the Galaxy Training Network (GTN) [56] which is a valuable asset to the accessibility and reusability of tools and workflows [59, 60].

If enough researchers and experts start using and contributing to the platform, the number and content of available analytical procedures could expand at the same pace as latest analytical methodologies are integrated to research processes. If a different platform fits best and is more widely used by ecological and biodiversity scientific communities in the end, the work done on Galaxy will not be lost as tools are easily transposable to other interfaces (*e.g.* scripts directly usable with R, Python, etc., translation of workflows to other workflow engines).

Galaxy is ready to use and has proved its efficiency and suitability in other research fields, including genomics and climate science [61, 62]. Galaxy-Ecology has implemented workflows for biodiversity data exploration, eDNA processing, general population and community metrics and models, ecoregionalisation, NDVI (Normalised difference vegetation index) computation with Sentinel-2 data among others [63] and tutorials for several of them are available on the GTN platform [64].

In addition to using existing tools, users may develop and upload entirely new tools and workflows to the Galaxy server in any computational language to make them accessible to all other users.

Galaxy is a participative platform and several ways to participate to Galaxy exist depending on one's skills, available time, and needs. Anyone can participate to the Galaxy-Ecology initiative by:

- Sharing datasets, histories and workflows;
- Giving feedback on servers, tools, and workflows;
- Sharing tools and workflows ideas (eventually with code) through Git issues;
- Asking for tool modifications through issues;
- Modifying existing tools or proposing new tools through GitHub or GitLab;
- Writing or contributing to a GTN tutorial on a specific functionality or a workflow on the Galaxy Training Network platform;
- Create learning pathways, a set of tutorials curated by community experts to form a coherent set of lessons around a topic, building up knowledge [65];
- Propose training events and help users in the utilisation of a workflow and tutorial.

Analyses are rarely computed only once. Any analysis with a generalisation potential is a suitable candidate to be Galaxy-fied. A methodological framework is presented in online supplementary material [66] at three levels depending on potential interests, computing language skills, and willingness to invest more or less time in the process: (i) 'user' relying on existing Galaxy tools and workflows to analyse data (lower time investment), (ii) 'developer' relying on existing and validated analytical procedure to develop Galaxy tools and workflows (highest time investment), and (iii) 'trainer' relying on existing Galaxy tools to share workflows and create training material (variable time investment).

Discussion and limitations

There are many best practices and recommendations existing for analytical procedures, data management, and computational code development. The levels of application of these

best practices fall within a continuum offering a range of possibilities from the sole sharing of processed and interpreted results with a brief description of methods to an executable paper published within a container and emulated virtual machine [17, 67]. Situated somewhere in between the aforementioned extremes, the atomisation – generalisation framework and the utilisation of the Galaxy platform might represent viable solutions offering a satisfactory level of best practices.

Atomisation and generalisation of computer codes can represent a relatively low investment strategy to attain certain levels of best practices such as transparency and reusability. It also carries advantages such as easier peer review, modularity of analytical procedures and, consequently, time savings. Indeed, applying the framework is not sufficient to attain the highest levels of best practices. For reproducibility and transparency, the management of the environment, software and package versions can be hard to maintain and record. For example, on a local computer a comprehensive tracking of input, outputs and codes requires meticulous management of folder structure in the environment. Additionally, non-code developers will be able to partially review the analytical procedure only if the workflow is clearly outlined in an adapted format (*e.g.* table, graphical representation). Accessibility and findability of the atomised and generalised analytical procedure is dependent of its proper sharing (*e.g.* persistent link, open repository). Galaxy can represent an easier gateway towards higher levels of best practice as sharing a complete, detailed and (re-)executable analytical procedure is facilitated through provenance tracking and automatic metadata enrichment. In comparison, many scientific workflow management systems, such as Snakemake, Nextflow or the R package Targets, operate from the command line. In ecology, numerous initiatives have tried to introduce such systems, starting with more user-friendly solutions. For example, the KNIME and Kepler systems with the CoESRA initiative (Collaborative Environment for Scholarly

Research and Analysis) in Australia; Taverna with the BioVeL initiative (Biodiversity Virtual e-Laboratory) in Europe; or very recently, the BON in a Box pipeline engine. These systems are more accessible to new users by offering a graphical interface while achieving high specificity [68 – 70]. However, good computer programming or scientific workflow management knowledge is still necessary to use these applications appropriately.

In comparison to the atomisation-generalisation framework, Galaxy can be rightfully seen as necessitating more time investment for scientists with programming experience as it requires to learn to use a new platform. Additionally, more effort may be required on Galaxy when an additional analytical step needs to be developed, but the Galaxy community can be an efficient crutch on which hard-pressed scientists can rely. Indeed, one can ask for help on the implementation of tools whether one knows computing languages and can share their code or not.

Conclusions

This article showcases a simple proposition to achieve best practices in analytical procedures with two plain guidelines: atomisation and generalisation. This straightforward framework represents a different manner to think and build analytical procedures; it doesn't require using a new technology or learning to use a new software. In terms of attaining higher levels of best practice, whether it is through the atomisation-generalisation framework, Galaxy, a combination of the two or otherwise, the optimal approach is to be determined by individuals depending on their interests, projects, and available resources. Relying on existing solutions as much as possible is, in our perspective, an efficient way to achieve a better understanding of best practices and their implications. Given the current environmental crisis, science has the major political and social responsibility to maintain good levels of transparency, reproducibility and efficiency.

Availability of supporting source code and requirements

Project name: Galaxy-Ecology tools

Project home page: https://github.com/galaxyecology/tools-ecology [71]

Software Heritage PID: swh:1:dir:2d6d04c76c640f6796c6bb27abfd42c63028d4ca

Operating system(s): Platform independent, installation using the Galaxy Tool Shed, notably through the Ecology section: https://toolshed.g2.bx.psu.edu/repository/browse_repositories_in_category?id=b4146bb7fe9b8726&message=&status=done)

Programming language: R, Python, XSLT

License: MIT

This has also been archived in Software Heritage [72]

The Workflow Hub dedicated project is available at [63] with related workflows [73-81]

Galaxy training materials "Ecology" topics are available at [64]: and associated workflows [84, 85].

Data Availability

Data shared to test Galaxy training materials on the topics "Ecology" are available in Zenodo [83]. Test data are also associated with the Galaxy-Ecology tools GitHub repository available at [71] in the test-data folder of each tool.

Declarations

Competing interests

The authors declare that they have no competing interests.

Funding

Funding were provided by the European Union through the Erasmus+ Gallantries project; the Agence Nationale de la Recherche through the 65 Million d'Observateurs and the IA-Biodiv projects; the French National Fund for Open Science through the OpenMetaPaper project; the European commission through the H2020 EOSC-Pillar, GAPARS projects, and Horizon Europe FAIRE EASE project; the GO FAIR initiative through the BiodiFAIRse Implementation Network; the Blue Nature Alliance; and the Antarctic and Southern Ocean Coalition. Finally, funding by the French Ministry of Higher Education and Research were provided for the "Pôle national de données de biodiversité" e-infrastructure.

Authors' contributions

C. R. drafted the article text, tables, and figures.

C. R. conceptualised the atomisation – generalisation framework with J.-B. M. and Y. L.B. while working on the development of Galaxy workflows.

J.-B. M. and Y. L.B. reviewed and helped rewrite many parts of the draft.

Y. R. and D. P. helped inspire and were invested in the early design of the article.

M. J. and P. S. tested and approved the appliance of the framework.

O. N., M. J., Y. R., M. E., B. B., A. F., H. R. and S. H. highly enhanced the quality of the redaction in both form and content at several stages of the draft.

H. R, S. H., B. B., A. F., and B. G. are involved in the Galaxy-E initiative and provided many advice on the redaction of the article and/or on the development of the initiative.

M. E. and G. M. are involved in Antarctic-oriented Galaxy tool and workflow development coordination.

C. B., R. L., A. M., Y. B., A. A., T. V. and V. C. developed scripts, tools and/or Galaxy workflows to contribute to the Galaxy-E initiative.

E. A. developed R scripts and apps used to integrate R Shiny apps as Galaxy interactive tools and initiate "Research Data management Galaxy tools".

E. M. and C. U. developed the first training materials for Galaxy-E.

E. T. worked on the use of the first Galaxy-E analysis.

M. D., G. L. and R. J. were coordinating the prefiguration of Galaxy-E through the 65 Millions d'Observateurs project.

Additionally, all authors reviewed and approved the article draft.

Acknowledgements

The authors want to thank Sandrine Pavoine for its highly relevant and helpful advice and reviews on both the content and the form of the article. Authors are thankful to Thimothée Poisot (recommender), Nick Isaac (reviewer) and one anonymous reviewer for their advice during the Peer Commnuity In review. Their help and suggestions on the structure and the content of the manuscript really helped to get the message of the article across in a more accessible manner.

References

1. Natural Environment Research Council (NERC). Most Wanted: Postgraduate Skills Needs in the Environment Sector. In: Living With Environmental Change report. 2010, 2012. https://webarchive.nationalarchives.gov.uk/ukgwa/20220214165229/https://nerc.ukri.org/skills/postgrad/policy/skillsreview/2012/. Accessed 26 October 2023.
2. Hampton SE, Jones MB, Wasser LA, Schildhauer MP, Supp SR, Brun J, Hernandez RR, Boettiger C, Collins SL, Gross LJ, Fernández DS, Budden A, White EP, Teal TK, Labou SG, Aukema JE. Skills and Knowledge for Data-Intensive Environmental Research. BioScience. 2017; https://doi.org/10.1093/BIOSCI/BIX025.

3. Emery NC, Crispo E, Supp SR, Farrell KJ, Kerkhoff AJ, Bledsoe EK, O'Donnell KL, McCall AC, Aiello-Lammens ME. Data Science in Undergraduate Life Science Education: A Need for Instructor Skills Training. BioScience. 2021; https://doi.org/10.1093/BIOSCI/BIAB107.
4. Ioannidis JPA. Correction: Why Most Published Research Findings Are False. PLoS Med. 2022; https://doi.org/10.1371/JOURNAL.PMED.1004085.
5. Fanelli D. Is science really facing a reproducibility crisis, and do we need it to? Proc Natl Acad Sci U S A. 2018; https://doi.org/10.1073/pnas.1708272114.
6. Plesser HE. Reproducibility vs. Replicability: A brief history of a confused terminology. Front Neuroinform. 2018; https://doi.org/10.3389/FNINF.2017.00076.
7. Cohen-Boulakia S, Belhajjame K, Collin O, Chopard J, Froidevaux C, Gaignard A, Hinsen K, Larmande P, Le Bras Y, Lemoine F, Mareuil F, Ménager H, Pradal C, Blanchet C. Scientific workflows for computational reproducibility in the life sciences: Status, challenges and opportunities. Future Gener Comput Syst. 2017; https://doi.org/10.1016/j.future.2017.01.012.
8. Wilkinson MD, Dumontier M, Aalbersberg IJ, Appleton G, Axton M, Baak A, Blomberg N, Boiten JW, da Silva Santos LB, Bourne PE, Bouwman J, Brookes AJ, Clark T, Crosas M, Dillo I, Dumon O, Edmunds S, Evelo CT, Finkers R, Gonzalez-Beltran A, Gray AJG, Groth P, Goble C, Grethe JS, Heringa J, t Hoen PAC, Hooft R, Kuhn T, Kok R, Kok J, Lusher SJ, Martone ME, Mons A, Packer AL, Persson B, Rocca-Serra P, Roos M, van Schaik R, Sansone SA, Schultes E, Sengstag T, Slater T, Strawn G, Swertz MA, Thompson M, Van Der Lei J, Van Mulligen E, Velterop J, Waagmeester A, Wittenburg P, Wolstencroft K, Zhao J, Mons B. Comment: The FAIR Guiding Principles for scientific data management and stewardship. Sci Data. 2016; https://doi.org/10.1038/sdata.2016.18.

9. Michener WK. Ten Simple Rules for Creating a Good Data Management Plan. PLoS Comput Biol. 2015; https://doi.org/10.1371/JOURNAL.PCBI.1004525.
10. Carroll S, Garba I, Figueroa-Rodríguez O, Holbrook J, Lovett R, Materechera S, Parsons M, Raseroka K, Rodriguez-Lonebear D, Rowe R, Sara R, Walker J, Anderson J, Hudson M. The CARE Principles for Indigenous Data Governance. Data Sci J. 2020; https://doi.org/10.5334/dsj-2020-043.
11. Araújo MB, Anderson RP, Barbosa AM, Beale CM, Dormann CF, Early R, Garcia RA, Guisan A, Maiorano L, Naimi B, O'Hara RB, Zimmermann NE, Rahbek C. Standards for distribution models in biodiversity assessments. Sci Adv. 2019; https://doi.org/10.1126/sciadv.aat4858.
12. Zurell D, Franklin J, König C, Bouchet PJ, Dormann CF, Elith J, Fandos G, Feng X, Guillera-Arroita G, Guisan A, Lahoz-Monfort JJ, Leitão PJ, Park DS, Peterson AT, Rapacciuolo G, Schmatz DR, Schröder B, Serra-Diaz JM, Thuiller W, Yates KL, Zimmermann NE, Merow C. A standard protocol for reporting species distribution models. Ecography. 2020; https://doi.org/10.1111/ecog.04960.
13. Archmiller AA, Johnson AD, Nolan J, Edwards M, Elliott LH, Ferguson JM, Iannarilli F, Vélez J, Vitense K, Johnson DH, Fieberg J. Computational Reproducibility in The Wildlife Society's Flagship Journals. J Wildl Manag. 2020; https://doi.org/10.1002/JWMG.21855.
14. Culina A, van den Berg I, Evans S, Sánchez-Tójar A. Low availability of code in ecology: A call for urgent action. PLoS Biol. 2020; https://doi.org/10.1371/JOURNAL.PBIO.3000763.
15. Minocher R, Atmaca S, Bavero C, McElreath R, Beheim B. Estimating the reproducibility of social learning research published between 1955 and 2018. R Soc Open Sci. 2021; https://doi.org/10.1098/RSOS.210450.

16. Ivimey-Cook ER, Pick JL, Bairos-Novak K, Culina A, Gould E, Grainger M, Marshall B, Moreau D, Paquet M, Royauté R, Sanchez-Tojar A, Silva I, Windecker S. Implementing Code Review in the Scientific Workflow: Insights from Ecology and Evolutionary Biology (pre-print). EcoEvoRxiv. 2023; https://doi.org/10.32942/X2CG64.
17. Grüning B, Chilton J, Köster J, Dale R, Soranzo N, van den Beek M, Goecks J, Backofen R, Nekrutenko A, Taylor J. Practical Computational Reproducibility in the Life Sciences. Cell Syst. 2018; https://doi.org/10.1016/j.cels.2018.03.014.
18. Mölder F, Jablonski KP, Letcher B, Hall MB, Tomkins-Tinch CH, Sochat V, Forster J, Lee S, Twardziok SO, Kanitz A, Wilm A, Holtgrewe M, Rahmann S, Nahnsen S, Köster J Sustainable data analysis with Snakemake [version 1; peer review: 1 approved, 1 approved with reservations]. F1000Res. 2021; https://doi.org/10.12688/f1000research.29032.1.
19. Mölder F, Jablonski KP, Letcher B, Hall MB, Tomkins-Tinch CH, Sochat V, Forster J, Lee S, Twardziok SO, Kanitz A, Wilm A, Holtgrewe M, Rahmann S, Nahnsen S, Köster J (2024) Snakemake (Version 8.22.0) https://github.com/snakemake/snakemake/releases/tag/v8.22.0.
20. Köster J, Rahmann S. Snakemake—a scalable bioinformatics workflow engine. Bioinformatics. 2012; https://doi.org/10.1093/bioinformatics/bts480.
21. Di Tommaso P, Chatzou M, Floden EW, Barja P., Palumbo E, Notredame C. Nextflow enables reproducible computational workflows. Nat. Biotechnol. 2017; https://doi.org/10.1038/nbt.3820.
22. Di Tommaso P, Chatzou M, Floden EW, Barja P., Palumbo E, Notredame C (2024) Nextflow (Version 24.04.4) https://github.com/nextflow-io/nextflow/releases/tag/v24.04.4.

23. The Galaxy Community. The Galaxy platform for accessible, reproducible and collaborative analyses: 2024 update. Nucleic Acids Res. 2024; https://doi.org/10.1093/nar/gkae410.

24. The Galaxy Community (2024) Galaxy (Version 24.1.2) https://github.com/galaxyproject/galaxy/releases/tag/v24.1.2.

25. Goble C, Cohen-Boulakia S, Soiland-Reyes S, Garijo D, Gil Y, Crusoe MR, Peters K, Schober D. FAIR Computational Workflows. Data Intell. 2020; https://doi.org/10.1162/dint_a_00033.

26. Lamprecht A-L, Garcia L, Kuzak M, Martinez C, Arcila R, Martin Del Pico E, Dominguez Del Angel V, van de Sandt S, Ison J, Martinez PA, McQuilton P, Valencia A, Harrow J, Psomopoulos F, Gelpi JL, Chue Hong N, Goble C, Capella-Gutierrez S. Towards FAIR principles for research software. Data Sci. 2019; https://doi.org/10.3233/ds-190026.

27. Munafò MR, Nosek BA, Bishop DVM, Button KS, Chambers CD, Percie Du Sert N, Simonsohn U, Wagenmakers EJ, Ware JJ, Ioannidis JPA. A manifesto for reproducible science. Nat Hum Behav. 2017; https://doi.org/10.1038/s41562-016-0021.

28. European Commission, Directorate-General for Research and Innovation. Cost-benefit analysis for FAIR research data : cost of not having FAIR research data. Publications Office of the European Union. 2018; https://doi.org/10.2777/02999.

29. Gomes DGE, Pottier P, Crystal-Ornelas R, Hudgins EJ, Foroughirad V, Sánchez-Reyes LL, Turba R, Martinez PA, Moreau D, Bertram MG, Smout CA, Gaynor KM. Why don't we share data and code? Perceived barriers and benefits to public archiving practices. Proc R Soc B. 2022; https://doi.org/10.1098/rspb.2022.1113.

30. Powers SM, Hampton SE. Open science, reproducibility, and transparency in ecology. Ecol Appl. 2019; https://doi.org/10.1002/eap.1822.

31. Lortie CJ. The early bird gets the return: The benefits of publishing your data sooner. Ecol Evol. 2021; https://doi.org/10.1002/ECE3.7853.
32. Jenkins GB, Beckerman AP, Bellard C, Benítez-López A, Ellison AM, Foote CG, Hufton AL, Lashley MA, Lortie CJ, Ma Z, Moore AJ, Narum SR, Nilsson J, O'Boyle B, Provete DB, Razgour O, Rieseberg L, Riginos C, Santini L, Sibbett B, Peres-Neto PR. Reproducibility in ecology and evolution: Minimum standards for data and code. Ecol Evol. 2023; https://doi.org/10.1002/ECE3.9961.
33. Michener WK, Jones MB. Ecoinformatics: Supporting ecology as a data-intensive science. Trends Ecol Evol. 2012; https://doi.org/10.1016/j.tree.2011.11.016.
34. Farley SS, Dawson A, Goring SJ, Williams JW. Situating Ecology as a Big-Data Science: Current Advances, Challenges, and Solutions. BioScience. 2018; https://doi.org/10.1093/BIOSCI/BIY068.
35. Jetz W, McGeoch MA, Guralnick R, Ferrier S, Beck J, Costello MJ, Fernandez M, Geller GN, Keil P, Merow C, Meyer C, Muller-Karger FE, Pereira HM, Regan EC, Schmeller DS, Turak E. Essential biodiversity variables for mapping and monitoring species populations. Nat Ecol Evol. 2019; https://doi.org/10.1038/s41559-019-0826-1.
36. Leroy B. Choosing presence-only species distribution models. J. Biogeogr. 2023; https://doi.org/10.1111/jbi.14505.
37. Boyd RJ, August TA, Cooke R, Logie M, Mancini F, Powney GD, Roy DB, Turvey K, Isaac NJB. An operational workflow for producing periodic estimates of species occupancy at national scales. Biol Rev. 2023; https://doi.org/10.1111/brv.12961.
38. EMBL Australia Bioinformatics Resource. Community Survey Report. 2013. https://www.embl-abr.org.au/news/braembl-community-survey-report-2013/. Accessed 7 Nov 2023.
39. Williams JJ, Teal TK. A vision for collaborative training infrastructure for bioinformatics. Ann N Y Acad Sci. 2017; https://doi.org/10.1111/NYAS.13207.

40. Larcombe L, Hendricusdottir R, Attwood T, Bacall F, Beard N, Bellis L, Dunn W, Hancock J, Nenadic A, Orengo C, Overduin B, Sansone S, Thurston M, Viant M, Winder C, Goble C, Ponting C, Rustici G. ELIXIR-UK role in bioinformatics training at the national level and across ELIXIR. F1000Res. 2017; https://doi.org/10.12688/f1000research.11837.1.
41. Touchon JC, McCoy MW. The mismatch between current statistical practice and doctoral training in ecology. Ecosphere. 2016; https://doi.org/10.1002/ECS2.1394.
42. Gownaris NJ, Vermeir K, Bittner MI, Gunawardena L, Kaur-Ghumaan S, Lepenies R, Ntsefong GN, Zakari IS. Barriers to Full Participation in the Open Science Life Cycle among Early Career Researchers. Data Sci J. 2022; https://doi.org/10.5334/DSJ-2022-002.
43. Fang FC, Casadevall A. Competitive Science: Is Competition Ruining Science? Infect Immun. 2015; https://doi.org/10.1128/IAI.02939-14.
44. Ellemers N. Science as collaborative knowledge generation. Br J Soc Psychol. 2021; https://doi.org/10.1111/BJSO.12430.
45. Keenan M, Cutler P, Marks J, Meylan R, Smith C, Koivisto E. Orienting international science cooperation to meet global “grand challenges”. Sci Public Policy. 2012; https://doi.org/10.1093/SCIPOL/SCS019.
46. Casajus N (2023). rcompendium: An R package to create a package or research compendium structure (Version 1.3) https://github.com/FRBCesab/rcompendium/releases/tag/v1.3.
47. Field B, Booth A, Ilott I, Gerrish K. Using the Knowledge to Action Framework in practice: a citation analysis and systematic review. Implement Sci. 2014; https://doi.org/10.1186/s13012-014-0172-2.

48. McIntire EJB, Chubaty AM, Cumming SG, Andison D, Barros C, Boisvenue C, Haché S, Luo Y, Micheletti T, Stewart FEC. PERFICT: A Re-imagined foundation for predictive ecology. Ecol Lett. 2022; https://doi.org/10.1111/ELE.13994.
49. The European Galaxy for Ecology instance. https://ecology.usegalaxy.eu. Accessed 21 Oct 2024.
50. The French Galaxy for Ecology instance. https://ecology.usegalaxy.fr. Accessed 21 Oct 2024.
51. WorkflowHub. https://workflowhub.eu. Accessed 21 Oct 2024.
52. Dockstore. https://dockstore.org. Accessed 21 Oct 2024.
53. The Research Data Management toolkit for Life Sciences – Elixir Europe. Tool Assembly – Galaxy. https://rdmkit.elixir-europe.org/galaxy_assembly. Accessed 21 Oct 2024.
54. Crusoe MR, Abeln S, Iosup A, Amstutz P, Chilton J, Tijanić N, Ménager H, Soiland-Reyes S, Goble C. Methods Included: Standardizing Computational Reuse and Portability with the Common Workflow Language. Commun ACM. 2022; https://doi.org/10.1145/3486897.
55. Soiland-Reyes S, Sefton P, Crosas M, Castro LJ, Coppens F, Fernández JM, Garijo D, Grüning B, La Rosa M, Leo S, Ó Carragáin E, Portier M, Trisovic A, Community R-C, Groth P, Goble C. Packaging research artefacts with RO-Crate. Data Sci. 2022; https://doi.org/10.3233/DS-210053.
56. Galaxy Training platform. https://training.galaxyproject.org. Accessed 21 Oct 2024.
57. Lai J, Lortie CJ, Muenchen RA, Yang J, Ma K. Evaluating the popularity of R in ecology. Ecosphere. 2019; https://doi.org/10.1002/ECS2.2567.
58. Galaxy Tool Shed. https://toolshed.g2.bx.psu.edu. Accessed 21 Oct 2024.
59. Batut B, Hiltemann S, Bagnacani A, Baker D, Bhardwaj V, Blank C, Bretaudeau A, Brillet-Guéguen L, Čech M, Chilton J, Clements D, Doppelt-Azeroual O, Erxleben A,

Freeberg MA, Gladman S, Hoogstrate Y, Hotz HR, Houwaart T, Jagtap P, Larivière D, Le Corguillé G, Manke T, Mareuil F, Ramírez F, Ryan D, Sigloch FC, Soranzo N, Wolff J, Videm P, Wolfien M, Wubuli A, Yusuf D, Taylor J, Backofen R, Nekrutenko A, Grüning B. Community-Driven Data Analysis Training for Biology. Cell Syst. 2018; https://doi.org/10.1016/j.cels.2018.05.012.

60. Hiltemann S, Rasche H, Gladman S, Hotz HR, Larivière D, Blankenberg D, Jagtap PD, Wollmann T, Bretaudeau A, Goué N, Griffin TJ, Royaux C, Le Bras Y, Mehta S, Syme A, Coppens F, Droesbeke B, Soranzo N, Bacon W, Psomopoulos F, Gallardo-Alba C, Davis J, Föll MC, Fahrner M, Doyle MA, Serrano-Solano B, Fouilloux AC, van Heusden P, Maier W, Clements D, Heyl F, Grüning B, Batut B. Galaxy Training: A powerful framework for teaching! PLoS Comput Biol. 2023; https://doi.org/10.1371/JOURNAL.PCBI.1010752.
61. Knijn A, Michelacci V, Orsini M, Morabito S. Advanced Research Infrastructure for Experimentation in genomicS (ARIES): a lustrum of Galaxy experience (pre-print). bioRxiv. 2020; https://doi.org/10.1101/2020.05.14.095901.
62. Serrano-Solano B, Fouilloux A, Eguinoa I, Kalaš M, Grüning B, Coppens F. Galaxy: A Decade of Realising CWFR Concepts. Data Intell. 2022; https://doi.org/10.1162/dint_a_00136.
63. WorkflowHub. PNDB (Pôle National de Données de Biodiversité) - workflows. https://workflowhub.eu/projects/19#workflows. Accessed 21 Oct 2024.
64. Galaxy Training platform. Ecology tutorials. https://training.galaxyproject.org/training-material/topics/ecology. Accessed 21 Oct 2024.
65. Galaxy Training platform. Learning pathways. https://training.galaxyproject.org/training-material/learning-pathways. Accessed 21 Oct 2024.

66. GitHub. ColineRoyaux – Galaxy templates repository. Methods - How to Galaxy-fy your analytical procedure? https://github.com/ColineRoyaux/Galaxy_Templates/blob/main/Methods/Methods%20-%20How%20to%20Galaxy-fy%20your%20analytical%20procedure_.md. Accessed 21 Oct 2024.
67. Strijkers R, Cushing R, Vasyunin D, De Laat C, Belloum ASZ, Meijer R. Toward executable scientific publications. Procedia Comput Sci. 2011; https://doi.org/10.1016/J.PROCS.2011.04.074.
68. Berthold MR, Cebron N, Dill F, Gabriel TR, Kötter T, Meinl T, Ohl P, Sieb C, Thiel K, Wiswedel B. KNIME: The Konstanz Information Miner. In: Preisach C, Burkhardt H, Schmidt-Thieme L, Decker R, editors. Data Analysis, Machine Learning and Applications. Studies in Classification, Data Analysis, and Knowledge Organization. Berlin, Heidelberg: Springer; 2008. p. 319-326. https://doi.org/10.1007/978-3-540-78246-9_38.
69. Hardisty AR, Bacall F, Beard N, Balcázar-Vargas MP, Balech B, Barcza Z, Bourlat SJ, Giovanni R, Jong Y, Leo F, Dobor L, Donvito G, Fellows D, Guerra AF, Ferreira N, Fetyukova Y, Fosso B, Giddy J, Goble C, Güntsch A, Haines R, Ernst VH, Hettling H, Hidy D, Horváth F, Ittzés D, Ittzés P, Jones A, Kottmann R, Kulawik R, Leidenberger S, Lyytikäinen-Saarenmaa P, Mathew C, Morrison N, Nenadic A, Hidalga AN, Obst M, Oostermeijer G, Paymal E, Pesole G, Pinto S, Poigné A, Fernandez FQ, Santamaria M, Saarenmaa H, Sipos G, Sylla KH, Tähtinen M, Vicario S, Vos RA, Williams AR, Yilmaz P. BioVeL: A virtual laboratory for data analysis and modelling in biodiversity science and ecology. BMC Ecol. 2016; https://doi.org/10.1186/S12898-016-0103-Y.
70. GEO BON - BON in a Box. https://boninabox.geobon.org/. Accessed 21 Oct 2024.
71. Galaxy Ecology Github: https://github.com/galaxyecology/tools-ecology. Accessed 10 December 2024.

72. Royaux C, Mihoub J-B, Jossé M, Pelletier D, Norvez O, and Reecht Y et al. (2024) Guidance framework to apply best practices in ecological data analysis: Lessons learned from building Galaxy-Ecology [computer software]. Software Heritage, https://archive.softwareheritage.org/swh:1:dir:2d6d04c76c640f6796c6bb27abfd42c63028d4ca;origin=https://github.com/galaxyecology/tools-ecology
73. Le Bras, Y., & Caon (LNE), Daniel. (2024). Evaluation IA-Biodiv workflow. WorkflowHub. https://doi.org/10.48546/WORKFLOWHUB.WORKFLOW.1181.1
74. Jossé, M., & Le Bras, Y. (2024). Obis biodiversity indicator on Asian pacific. WorkflowHub. https://doi.org/10.48546/WORKFLOWHUB.WORKFLOW.662.1
75. Jossé, M., & Le Bras, Y. (2024). Boulder fields indicators. WorkflowHub. https://doi.org/10.48546/WORKFLOWHUB.WORKFLOW.661.1
76. Le Bras, Y. (2024). SPIPOLL MMOS GAPARS crowdsourcing results. WorkflowHub. https://doi.org/10.48546/WORKFLOWHUB.WORKFLOW.660.1
77. Le Bras, Y., Seguineau, P., & Royaux, C. (2024). Ecoregionalization on Antarctic sea. WorkflowHub. https://doi.org/10.48546/WORKFLOWHUB.WORKFLOW.658.1
78. Le Bras, Y., Royaux, C., & Jossé, M. (2024). Biodiversity data exploration tutorial. WorkflowHub. https://doi.org/10.48546/WORKFLOWHUB.WORKFLOW.656.1
79. Le Bras, Y., & Royaux, C. (2024). Obitools eDNA metabarcoding. WorkflowHub. https://doi.org/10.48546/WORKFLOWHUB.WORKFLOW.655.1
80. Le Bras, Y. (2024). GBIF data Quality check and filtering workflow Feb-2020. WorkflowHub. https://doi.org/10.48546/WORKFLOWHUB.WORKFLOW.404.1
81. Le Bras, Y., & Royaux, C. (2024). Population and community metrics calculation from Biodiversity data. WorkflowHub. https://doi.org/10.48546/WORKFLOWHUB.WORKFLOW.49.2

82. Bras Y, Royaux C (2020) Population and community metrics calculation from Biodiversity data [workflow], https://doi.org/10.48546/workflowhub.workflow.49.2
83. Training materials on "ecology" in Zenodo. https://zenodo.org/communities/galaxy-training/records?q=yvan&f=resource_type%3Adataset&l=list&p=1&s=10&sort=bestmatch. Accessed 10 December 2024.
84. Seguineau, P. (2024). Ecoregionalization workflow (Part 1). WorkflowHub. https://doi.org/10.48546/WORKFLOWHUB.WORKFLOW.1208.
85. Seguineau, P. (2024). Ecoregionalization workflow (Part 2). WorkflowHub. https://doi.org/10.48546/WORKFLOWHUB.WORKFLOW.1209.1

Table 2 Click here to access/download;Table;supp_file_tab2.docx

Table 2 - Illustration of how the atomisation-generalisation framework and Galaxy implements and conforms to best practice.

		Atomised-generalised code	Galaxy
Reproducibility and transparency	Environment, software and package versions	Can be indicated but possibly hard to manage<br>Can also be set as an output of the analysis (*e.g.* session info)<br>Packages written in each coded elementary step or using a versioning system such as Conda	Entirely packaged with Conda package manager and BioContainers<br>Possibility to store analytical procedures as containers for persistent execution
	Inputs and parameters	One must keep track of different parametrisation and input settings at each computation	Automatically tracked and shareable with the "Galaxy history"
	Peer-review	Organisation of the analytical procedure reviewable by non-code developers<br>Code developers might be able to detect errors as it is easier in shorter scripts<br>Transparency over the development process achievable through Git	Reviewable "Galaxy history" and re-executable workflow<br>Continuous peer-reviewed of tools with open-source code<br>Transparency over the development process through Git<br>The workflows can be reviewed by the Intergalactic Workflow Commission (IWC) for best practices
	Output provenance	Can be tracked and reproduced in some cases	Tracked with the "Galaxy history" and reproducible with workflow
FAIR principles	Findable	If properly shared	Web-based solution<br>Unified system for data and software citation and attribution<br>Tools can be made available on several servers<br>Tools can be linked to tools registries and annotated with different ontologies<br>Annotated workflows findable on WorkflowHub [51] and Dockstore [52]
	Accessible	If properly shared	Free distribution of tools via the Galaxy ToolShed and workflows via WorkflowHub and Dockstore under an open-source licence
	Interoperable	When properly generalised, different elementary steps should be useable in interaction with each other	Use different software, computational language and library versions on a single platform with the Conda package management system<br>Workflows exportable in JSON and shareable through several standards (*e.g.* Common Workflow Language [54] and Research Object Crate [55])
	Reusable	Generalised elementary steps are reusable and adaptable with different analytical procedure, parametrisation and/or inputs	Tools, histories and workflows are re-executable, reusable and adaptable with different analytical procedure, parametrisation and/or inputs. Open-source code can be used outside of a Galaxy server
Technical and knowledge gaps	Understandability	The analytical procedure is clearer when properly atomised	Tools interface, workflow annotations, help sections and tutorials are a valuable help
	Teaching opportunities	Learning the analytical procedure design separately from computing languages, giving structure to trainees<br>Reusability of elementary steps for trainees	Experimenting with intricate analyses without computer code first<br>Tutorials and videos from Galaxy Training Network [56]<br>Galaxy community
	Computing capacity	Need for a computation cluster if large data or demanding algorithm	HPC (High Performance Computing) through an interface<br>Bulk (meta)data manipulation
Collaboration and attribution	Analysis design and development	Achievable through collaborative code-editing applications	With anyone through a Galaxy server
	Citation	Easy reuse of openly shared elementary steps could lead to higher citation rates	Each tool, workflow, and tutorial are provided with a unique identifier for proper attribution and citation

Figure 1

Click here to access/download;Figure;Fig1.tif

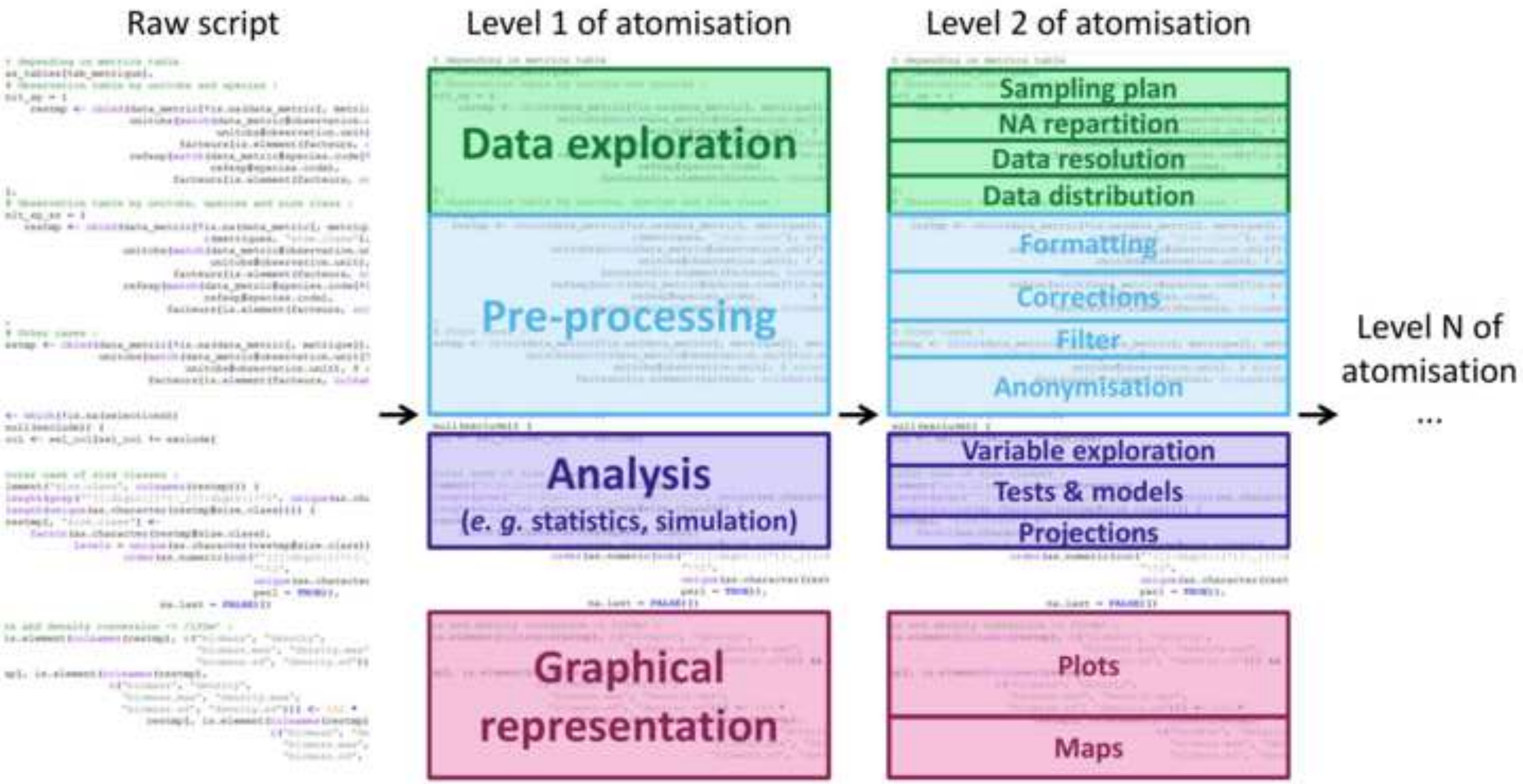

Figure 2

Click here to access/download;Figure;fig2.tif

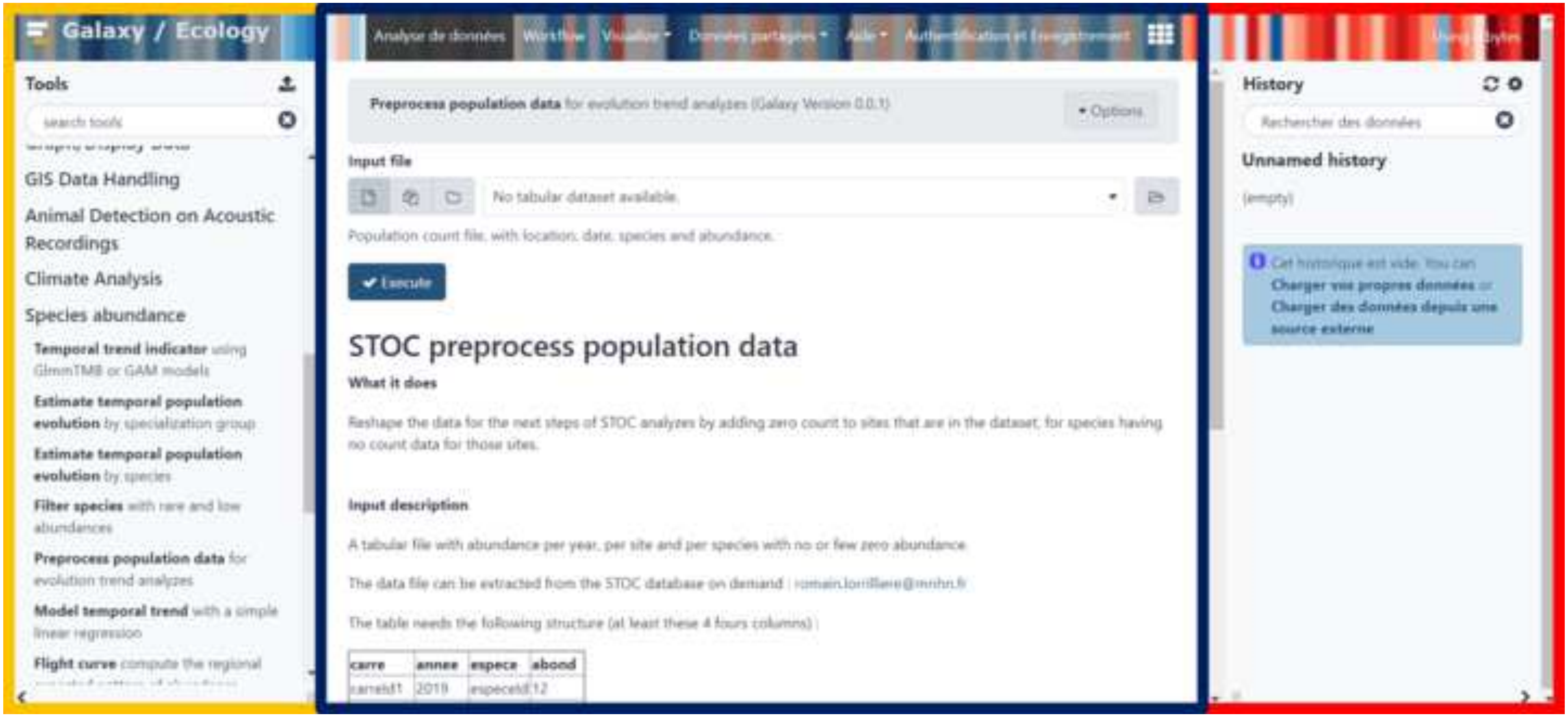

Figure 3

Click here to access/download;Figure;Fig3.png

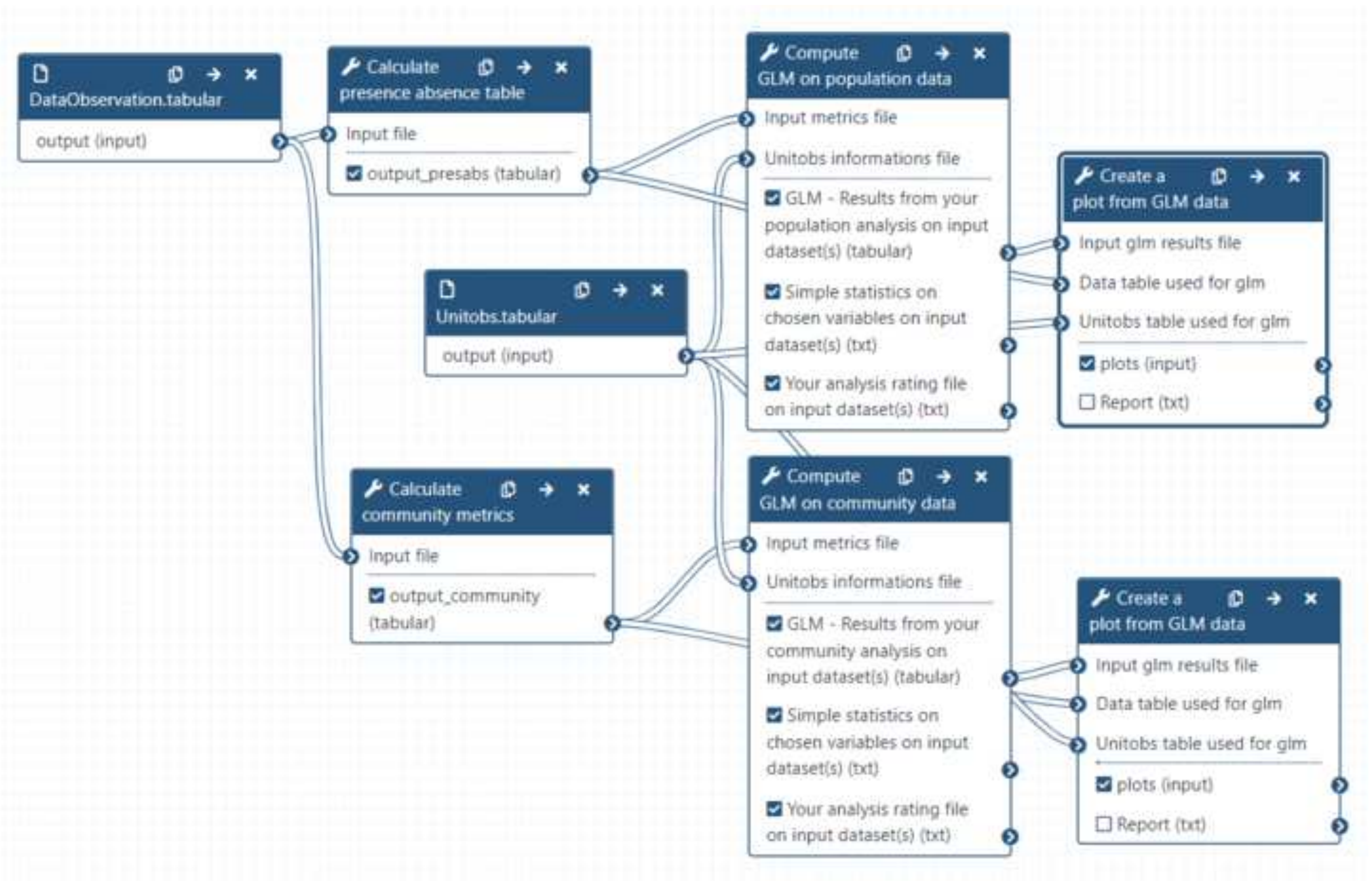

Personal Cover

Click here to access/download;Personal Cover;Cover_letter_GS.pdf

Coline Royaux

Sorbonne Université, Muséum national d'Histoire naturelle (UMR BOREA, UAR PatriNat)

29900 Concarneau – France

October 07, 2024

Dear Editors,

Please find enclosed a manuscript we would like to submit as a technical note to *GigaScience.* It is entitled "Guidance framework to apply best practices in ecological data analysis: Lessons learned from building Galaxy-Ecology". This manuscript has passed the *Peer Community In Ecology* recommendation process. The review process and the recommendation written by Timothée Poisot is available at this link: https://doi.org/10.24072/pci.ecology.100694. Additionally, we have been in touch with Scott Edmunds to enquire about discount for Galaxy-related papers that have been presented during the Galaxy Community Conference. He confirmed to us that a discount is applicable to this publication. Indeed, we have presented this work at several occasions at GCC since 2016 (see table on next page).

Striving to improve the practice of science to ensure transparency, efficiency and collaboration is a responsibility for all scientists, especially in the field of ecology and environment given the current crisis. Despite a rich conceptualisation of good practice available for scientific data and analysis, practice change in the ecological sciences still seems insufficient. The necessary change in practices needs to be facilitated by straightforward and simple guidelines. In this article, we discuss a practical framework we elaborated to maximise the reproducibility, understandability, accessibility, interoperability and reusability of analytical processes, which can be summarised in two words: atomisation and generalisation.

Atomisation is the decomposition of an analytical process into elementary steps and generalisation is the transformation of these elementary steps to make them reusable in different contexts and with different data formats. When applied correctly, these simple guidelines can be notably beneficial to peer-review, collaborative work and teaching.

As the framework was conceptualised in particular during the work on the enrichment of the Galaxy-Ecology initiative, the platform is also introduced as a demonstration of further good practices. In addition, this demonstration can help readers to better capture how to apply the atomisation – generalisation framework and even, inspire them to go further in improving their practice.

We trust this work would be of great relevance to the ecoinformatics research community and we hope that you will find our manuscript suitable for publication in *GigaSicence*.

Kind regards,

Coline Royaux, c/o the authors

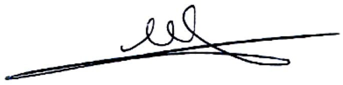

Year	Presenter(s)	Link
2016	Yvan Le Bras	https://gcc16.sched.com/event/7Zgd/65-millions-of-observers
2017	Valentin Chambon<br>Thimothée Virgoulay	https://gcc2017.sched.com/event/B5xf/galaxy-e-towards-an-accessible-reproducible-and-transparent-data-analysis-and-management-universe-dedicated-to-ecology
2018	Yvan Le Bras	https://gccbosc2018.sched.com/event/Eizd/galaxy-e-a-first-step-towards-collaborative-data-analysis-by-citizens
2019	Yvan Le Bras	https://gcc2019.sched.com/event/R1sX/poster-46-a-galaxy-e-project-2019-news
2020	Coline Royaux	https://bcc2020.sched.com/event/csup/essential-biodiversity-variables-on-galaxy-implementing-the-pampa-application<br><br>https://bcc2020.sched.com/event/csum/producing-biodiversity-indicators-from-citizen-science-projects-update-of-birds-and-bats-monitoring-schemes-on-galaxy-e
2021	Coline Royaux	https://gcc2021.sched.com/event/jm7q/french-ebv-operationalization-pilot-where-galaxy-can-help-biodiversity
2022	Yvan Le Bras	https://gcc2022.sched.com/event/135EA/galaxy-e-ecology-oriented-galaxy-initiative-a-2022-update
2023	Marie Jossé	https://docs.google.com/document/d/1xJVtNlN3D0yEZceoPb0CcdxtXP0Rx0FUo_ixU38wqlA/edit#heading=h.26t5p5i6044m p. 21 & p. 37
2024	Marie Jossé	https://galaxyproject.org/events/gcc2024/training/ecology/

Graphical Abstract

Click here to access/download;Graphical Abstract;Graph_abstract.tif

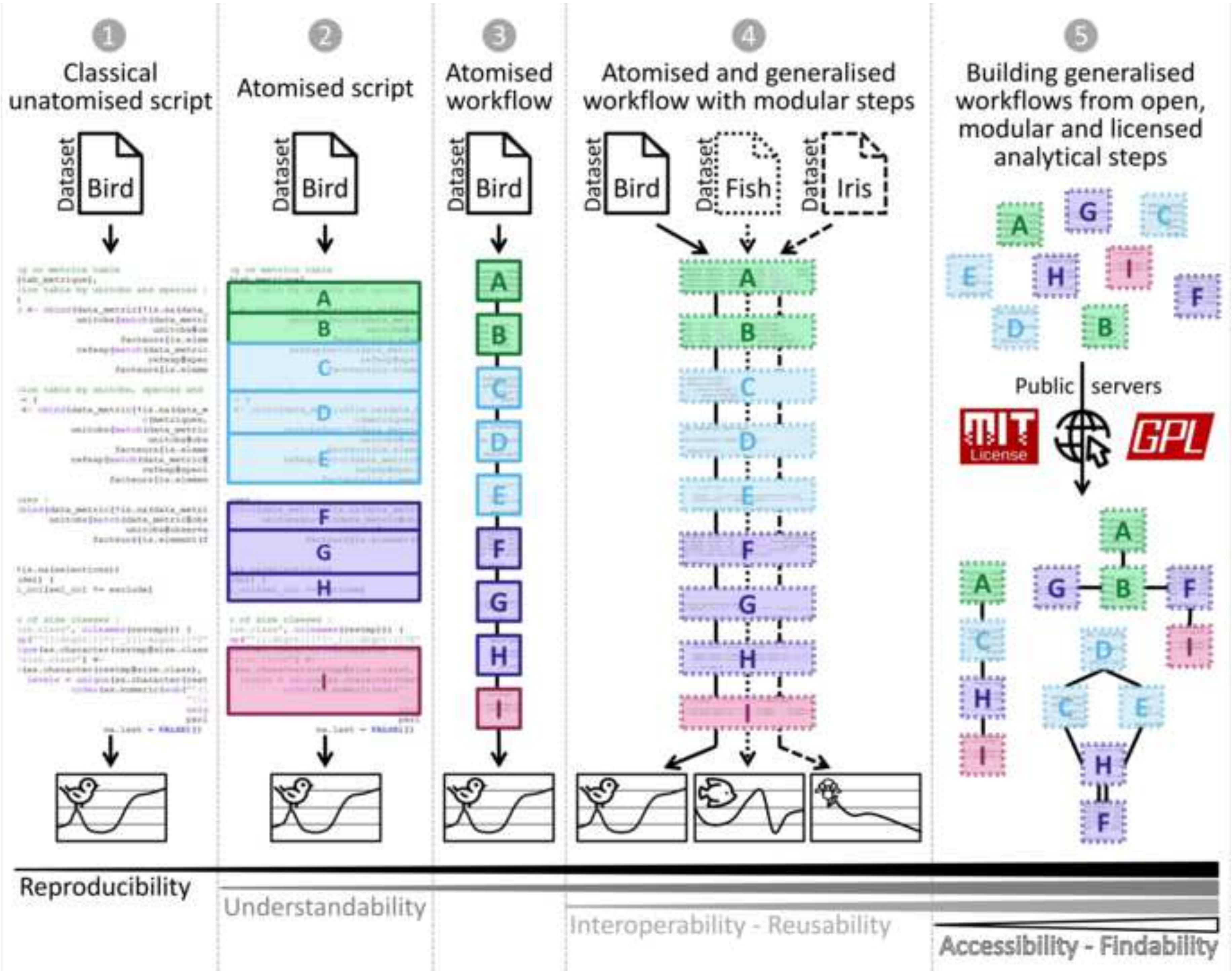

Supplement: giae122_GIGA-D-24-00463_Revision_1 [file giae122_giga-d-24-00463_revision_1.pdf]
