# Supplementary material for: Guidance framework to apply best practices in ecological data analysis: lessons learned from building Galaxy-Ecology
Source: Gigascience. 2025 Feb 12;14:giae122. doi: 10.1093/gigascience/giae122 (PMC11816794; doi:10.1093/gigascience/giae122)
Supplement: giae122_Response_to_Reviewer_Comments_Original_Submission [file giae122_response_to_reviewer_comments_original_submission.pdf]

Author's Response To Reviewer Comments

Comments from Nicole Nogoy

- Please list the following for the Galaxy-E platform:
Project name: e.g. My bioinformatics project
Project home page: e.g. https://github.com/ISA-tools
Operating system(s): e.g. Platform independent
Programming language: e.g. Java
Other requirements: e.g. Java 1.3.1 or higher, Tomcat 4.0 or higher
License: e.g. GNU GPL, FreeBSD etc.
RRID: if applicable, e.g. RRID: SCR_014986
Also; cite the Workflow Hub DOI.
- Response : It has been added.
- please also include information on supporting data used to demonstrate the Galaxy-E platform here listing accessions.
- Response : It has been added.
- Please add abbreviations here used more than once in alphabetical order.
- Response : There are no abbreviations in the text if we are not mistaken.
